# An economic evaluation of triage tools for patients with suspected severe injuries in England

**DOI:** 10.1186/s12873-021-00557-6

**Published:** 2022-01-11

**Authors:** Daniel Pollard, Gordon Fuller, Steve Goodacre, Eveline A. J. van Rein, Job F. Waalwijk, Mark van Heijl

**Affiliations:** 1 School of Health and Related Research, University of Sheffield, Sheffield, UK; 2 Department of Traumatology, University Medical Center Utrecht, Utrecht, the Netherlands

**Keywords:** Major trauma, Severe injuries, Triage tools, Economic evaluation

## Abstract

**Background:**

Many health care systems triage injured patients to major trauma centres (MTCs) or local hospitals by using triage tools and paramedic judgement. Triage tools are typically assessed by whether patients with an Injury Severity Score (ISS) ≥ 16 go to an MTC and whether patients with an ISS < 16 are sent to their local hospital. There is a trade-off between sensitivity and specificity of triage tools, with the optimal balance being unknown. We conducted an economic evaluation of major trauma triage tools to identify which tool would be considered cost-effective by UK decision makers.

**Methods:**

A patient-level, probabilistic, mathematical model of a UK major trauma system was developed. Patients with an ISS ≥ 16 who were only treated at local hospitals had worse outcomes compared to being treated in an MTC. Nine empirically derived triage tools, from a previous study, were examined so we assessed triage tools with realistic trade-offs between triage tool sensitivity and specificity. Lifetime costs, lifetime quality adjusted life years (QALYs), and incremental cost-effectiveness ratios (ICERs) were calculated for each tool and compared to maximum acceptable ICERs (MAICERs) in England.

**Results:**

Four tools had ICERs within the normal range of MAICERs used by English decision makers (£20,000 to £30,000 per QALY gained). A low sensitivity (28.4%) and high specificity (88.6%) would be cost-effective at the lower end of this range while higher sensitivity (87.5%) and lower specificity (62.8%) was cost-effective towards the upper end of this range. These results were sensitive to the cost of MTC admissions and whether MTCs had a benefit for patients with an ISS between 9 and 15.

**Conclusions:**

The cost-effective triage tool depends on the English decision maker’s MAICER for this health problem. In the usual range of MAICERs, cost-effective prehospital trauma triage involves clinically suboptimal sensitivity, with a proportion of seriously injured patients (at least 10%) being initially transported to local hospitals. High sensitivity trauma triage requires development of more accurate decision rules; research to establish if patients with an ISS between 9 and 15 benefit from MTCs; or, inefficient use of health care resources to manage patients with less serious injuries at MTCs.

**Supplementary Information:**

The online version contains supplementary material available at 10.1186/s12873-021-00557-6.

## Background

Major trauma is a significant problem worldwide, with a World Health Organisation report identifying that injuries were responsible for 9% of all deaths in 2012 [1]. Systems and interventions to improve the outcomes of patients with injuries represent a key area in which public health can be improved worldwide.

Major trauma centres (MTCs), which concentrate severely injured patients in specialist centres, were introduced in England in 2012. Similar systems have been in use in some regions of the USA for many decades. In MTC systems if patients are suspected to be severely injured then paramedics will bypass local hospitals, if these are closer than the MTC, and the MTC will be pre-alerted to allow activation of a specialist major trauma team for resuscitation and initial management. Evidence from the USA shows that patients who have an injury severity score (ISS) of 16 or more would, on average, have better outcomes if they were treated at a major trauma centre [2–6]. Consequently, severe injuries are often defined in the literature as patients whose ISS was 16 or more. However, ISS can only be derived after the patient has been diagnosed and treated, therefore it is not always clear which hospital the paramedics should decide to transport the patients to. Furthermore, there is a definition of severe injuries that defines severe injuries as those injuries that would benefit from care that is only available at MTCs in a US setting [7]. As this is not yet widely used in the literature to estimate the benefits of MTC care, we use the ISS definition of MTC need.

van Rein et al. conducted a systematic review of studies assessing the effectiveness of triage tools in MTC systems [8]. This study found that no study which had a high methodological quality produced a tool that had adequate performance. Consequently, there is clinical value in developing, testing and implementing triage tools for patients with suspected major trauma that can be applied by paramedics when initially assessing an injured patient. Existing triage tools consist of physiological, anatomical, injury characteristic, and injury mechanism variables. Sensitivity and specificity are dependent on which variables are included in the triage tool and the cut-off level chosen for constituent variables. Any triage tool, will trade-off the number of true positive cases correctly admitted to major trauma centres (sensitivity), with the number of true negative cases correctly transported to local hospitals (specificity). However, it is uncertain where the optimal balance of sensitivity and specificity lies as the use of MTCs is associated with improved outcomes for severely injured patients, but also costs more.

The aim of this paper is to conduct a cost-utility analysis of several plausible major trauma triage tools from the perspective of a UK decision maker. Secondary outcomes of the decision analytic model include system flows of patients throughout the model, as this will influence which tools are feasible.

## Methods

### Design

The decision problem was “which combination of sensitivity and specificity on a plausible receiver operator curve is the most cost-effective for patients presenting with suspected major trauma to paramedics in the UK?”. A cost-utility analysis was preformed using a probabilistic decision analytic model. A decision analytic model was chosen as this allows us to synthesise evidence from multiple data sources to assess multiple major trauma triage rules.

Our analysis only considers outcomes for patients who were injured outside of the MTC’s local area for two reasons. Firstly, patients who live closest to an MTC will usually go to the MTC regardless of the severity of their injury. However, if a patient is thought to be severely injured, the MTC will be pre-alerted. Secondly, the available effectiveness evidence for MTCs appears to treat all patients who live closest to an MTC as having been treated at an MTC regardless of whether the trauma team were pre-alerted. Consequently, there is no trade-off between cost and effectiveness that can be assessed in our analyses for patients who were injured closest to an MTC without new clinical studies.

Our analysis uses ISS as the reference standard for MTC care. This is because the evidence available on the effectiveness of MTCs only uses ISS to define who benefits from MTC care. Without any evidence supporting how MTC’s effect these outcomes for patients meeting / not meeting other criteria, such as the Lerner et al. criteria [7], economic analyses can only be designed to assess triage tools based on how well they predict whether a patient has an ISS ≥ 16.

In line with guidance from the English and Welsh (two constituent nations of the UK) decision maker, the National Institute for Health and Care Excellence (NICE): we undertook a cost-utility analysis; our analyses had a lifetime horizon; an NHS and personal social services perspective was taken; and, future costs and QALYs were discounted at 3.5% per annum [9].

### Interventions

Nine triage tools were examined, based on Newgard et al in which triage tools were derived by statistically analysing a retrospective cohort study conducted at 6 sites in the Western US between January 2006 and December 2008 [10]. This study was selected as it fit nine triage tools to one dataset, so represented feasible trade-offs between sensitivity and specificity for any new triage tool that may be developed in the UK. No similar study conducted in England was known to the authors. Clinical input from clinicians in an English Major Trauma Network was that the trade-off between the triage tools in Newgard et al. were likely to be similar for any newly developed triage tools in England [10]. These analyses produced nine triage tools for which, the reported sensitivities and specificities for each tool were:

- Sensitivity 99.8%, Specificity 2.5%
- Sensitivity 94.8%, Specificity 18.7%
- Sensitivity 90.4%, Specificity 58.4%
- Sensitivity 87.5%, Specificity 62.8%
- Sensitivity 74.6%, Specificity 65.7%
- Sensitivity 69.8%, Specificity 70.1%
- Sensitivity 64.2%, Specificity 76.1%
- Sensitivity 57.0%, Specificity 80.0%
- Sensitivity 99.8%, Specificity 88.6%

We assumed that the sensitivity and specificity values for each triage tool represented the final triage decision, combining the diagnostic accuracy of the triage tool and the application of judgement by the on-scene paramedics. This means that the sensitivity is the probability that a patient with an ISS ≥ 16 is sent to an MTC and the specificity is the probability that a patient with an ISS < 16 is sent to a local hospital. A recent analysis of a Dutch triage tool in an English data set produced a received-operator curve that would result in similar triage tool sensitivity and specificity as observed by Newgard et al. [11].

### Population

The model was populated with simulated patients’ representative of injured patients presenting to an English trauma system. To generate these characteristics we obtained access to baseline demographic and clinical data from a recent prehospital major trauma triage study by van Rein et al., using the data collected in the Central Netherlands region [12]. This provided a high quality data set, which we expected to be similar to patients in the UK, as a recent review of major trauma systems identified that there were many similarities between the major trauma system in the UK and the Netherlands and the implementation of major trauma systems in these two countries had produced similar outcomes [13]. From this data we obtained patient’s Age, Gender, ISS, Glasgow Coma Scale (GCS) and trauma type. Means and standard deviations of this data is summarised in Table 1. Information on how this data was used to sample patient characteristics in the model are provided in the supplementary material. Alternative English data sources were explored for the model population; however these sources were not appropriate as they were not representative of patients presenting with suspected major trauma to paramedics.

**Table 1:** A summary of the simulated characteristics of the patients included in the model

Characteristic	Mean	SD / n/N	Source
Age	46.8	21.3	Patients with complete Age, Gender, ISS, GCS and trauma type data in Van Rein et al. [12]
Percentage Male	58.3%	2887/4720	
ISS	5.2	7.2	
Percentage with an ISS ≥ 16	9.1%	428/4720	
GCS	14.4	1.9	
Percentage with blunt trauma	98.2%	4637/4720	

*SD* standard deviation, *ISS* injury severity score, *GCS* Glasgow Coma Scale

### Modelling approach

We developed a lifetime patient-level decision tree, followed by a discrete event simulation to model patient flows through an English MTC system. The model estimated patient’s outcomes, costs incurred, and quality adjusted life years (QALYs) accrued for patients with suspected severe injuries. Conceptual modelling, informed by a previously published model by Newgard et al and consultation with subject experts informed the final model design [14]. We have taken a patient-level approach, rather than the previously adopted cohort modelling approach, for two reasons. Firstly, we can predict the risk of death using a validated 30-day probability of survival equation, developed by the Trauma Audit and Research Network (TARN), in an English population [15, 16]. Secondly, when actual tools are assessed in English populations, this model can be updated to include the triage tool itself and the original population in which the tool was assessed. This approach would allow an analyst to include any correlations that may exist between a triage tool’s ability to predict major trauma centre need and the probability of death, as predicted by the TARN survival equation.

### Model structure

**Fig. 1:**
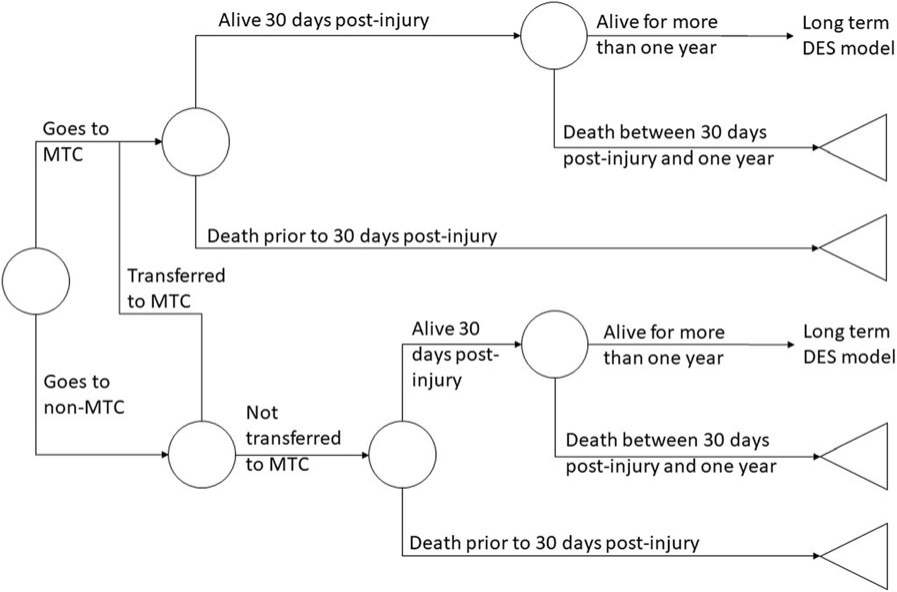
The model structure

The model structure is presented in Fig. 1. When a patient enters the model the triage tool determines whether the initial decision is to send them to the MTC or local hospital. Patients who go to an MTC, may be sent to a local hospital initially to receive urgent medical care and patients who initially go to a local hospital may be referred onwards to an MTC. Any patient who receives care at an MTC will gain the full benefit from MTC care, but all patients who initially go to a local hospital will incur costs for an additional ambulance callout.

Post-admission, each patient’s probability of survival within 30 days is estimated. Patients who survive up until 30 days post-injury, have their probability of survival up to one-year post-injury estimated. Patients with an ISS of 16 or more who received MTC care will have lower probabilities of death than the same patient who did not receive MTC care. ISS was chosen as the criteria by which patients would benefit from MTC care, as whilst there are new definitions of MTC need there is little evidence linking these definitions to mortality or QALYs.

Patients who survive up to one-year post-injury, enter a long-term discrete event simulation. In this simulation, their life expectancy is estimated using information on the increased risk of death faced by patients with a history of major trauma and general population mortality data.

This model was developed in R v4.0.2 [17]..

### Data - probability of events and effectiveness of MTCs

We used the same evidence as the previously published Newgard et al economic model for the effectiveness of MTCs on patient outcomes. These studies are analyses of large cohort studies in a North American setting [2–4]. Data on the probability of ambulance transfer and the probability of death between 30 days post-injury and 1 year post-injury also used the same evidence as the Newgard et al model, as no UK specific evidence was available to update these parameters [14]. For the probability of death within 30 days of injury we used the English based TARN survival prediction model and for the general population mortality we use UK life tables [15, 18]. We used the 2006 TARN survival prediction model in our base case economic model, as our patient cohort did not have information on comorbidities which are required in the 2015 TARN survival equation [15, 16].

### Data – quality adjusted life years

QALYs are calculated by multiplying the number of years that a patient has lived by a utility score, which is a number that reflects patient’s health related quality of life and is anchored on two points with 1 being equivalent to perfect health and 0 being equivalent to death. The utility parameters used in the model are provided in Table 2. Our utilities are from Ahmed et al, which is a survey in which 154 patients, whose ISS was 9 or more, completed the EQ-5D-5L questionnaire at an English MTC 1 year post-injury [21]. Utility was presented in the following ISS subgroups: ISS = 9 (*n* = 55), ISS = 10–14 (*n* = 26), ISS ≥ 16 (*n* = 73). This study was chosen as the analysis matched NICE’s reference case for producing utility values and the EQ-5D responses came from a relatively large sample in a UK population [30].

There was no evidence in this study that utility varied by ISS score, as the 95% confidence intervals for the mean utility score across the ISS subgroups overlapped with each other. For patients with an ISS of 9 or more we applied this utility multiplicatively to age-gender matched utilities for the UK general population [31]. In the multiplicative approach, people with an ISS of 9 or more will have their utility changed from the general population value by a constant proportion. For example, if the calculated utility multiplier was 0.8 (i.e. these patient would have 80% of the utility of the general population) and utility of a similar patient in the general population was expected to be 0.7 given the patients’ age and gender, then the patient’s utility would be 0.8*0.7 = 0.56.

For patients with an ISS of less than 9 we assumed that their injury did not have long term effects on their utility, as there was little evidence that there were long term effects on their utility.

### Data - costs

The costs in the model reflect English practice and are provided in Table 2. All costs are in 2017/18 prices. Costs from previous years were inflated to 2017/18 prices using the HCHS Pay and Prices inflation index [32]. Other costs incurred within the first 6 months post-injury were obtained from UK based studies and sources [23–26]. [John Nicholl, personal communication] After 6 months we used English health care costs incurred by the general population, according to their age and gender, and the data from Delgado et al to calculate the increased long term health care costs incurred by each patient due to their history of suspected major trauma in our model [28, 29].

### Outcome measures

The primary outcome measure of the model was the incremental cost-effectiveness ratio (ICER). Key secondary outcomes of the model included: life expectancy; discounted costs; discounted QALYs; the number of patients who died prior to discharge; the number of patients who died between discharge and one-year post-injury; and, the number of patients sent to an MTC per 100,000 cases of suspected major trauma.

The ICERs between the strategies were calculated as difference in cost / difference in QALYs. As we have a decision problem with multiple strategies, a full incremental analysis was undertaken in line with the NICE methods guide [9].. In this approach, strategies are ordered by their effectiveness (measured in QALYs). Any tools that are dominated (they produce less QALYs at a higher cost than another tool) or extendedly dominated (a combination of two other tools can produce the same QALYs at a lower cost) were removed from consideration. ICERs were then calculated comparing each remaining tool, to the next least effective remaining tool (except the least effective tool which acts as the comparator).

### Interpreting the ICER

The maximum acceptable ICER (MAICER), is the amount of money that a decision-maker is willing to pay to gain 1 additional QALY. NICE’s MAICER is usually considered to be £20,000 per QALY, but may increase to £30,000, as detailed in the NICE methods guide [9]. In a full incremental analysis, the most effective tool with an ICER below the decision maker’s MAICER is the cost-effective tool.

### Scenario analyses

A base case deterministic analysis was performed, where all parameters are set to mean values. In order to account for the uncertainty in model inputs a probabilistic sensitivity analysis (PSA) was conducted. In the PSA every parameter is randomly drawn from its assigned distribution and the model results (for all outcomes) for this set of parameters was recorded (see Additional file 1: Appendix). All model outcomes, excluding ICERs, were calculated by obtaining the mean value of the model outputs across all PSA results. ICERs were calculated from the mean costs and mean QALYs for each triage tool. If the PSA and deterministic results differ, the PSA results are the results to be believed as these consider all uncertainty in the input parameters [33]. We assessed the stability of our model results with respect to the number of patients (assessed visually) and number of PSA runs (assessed using the Hatswell et al method) [33]. We found that 25,000 simulated patients and 2000 PSA runs produced stable results to allow an economic analysis to be conducted (see Additional file 1: Appendix).

We conducted three scenario analyses to explore the robustness of model assumptions.

In the first scenario analysis we used the 2015 TARN survival equation. As our cohort had no information on patient’s Charlson Comorbidity Index (CCI), we assumed that the simulated patients in our model were in the same risk category as people with missing CCI in the TARN dataset [16].

In the second set of scenario analyses, we explored the benefit of MTC care to patients with an ISS between 9 and 15 inclusive. These patients incur costs for going to an MTC in England implying there may be a belief by payers that these patients would benefit from MTC care.

In the final set of scenario analyses we varied the cost of MTC care, as the cost of MTC care in England is reviewed regularly.

## Results

### Base case analysis

The results of the deterministic base case analysis are given in Table 3. All ICERs are in excess of £30,000 per QALY gained, consequently they are above the upper limit of the ICER that NICE would consider acceptable, meaning that the cost-effective strategy is the least sensitive triage tool [9]. The PSA results are given in Table 3. The PSA results, in terms of the ICERs and the tools which are dominated or extendedly dominated, are very different to the deterministic results. Consequently all conclusions and scenario analyses are based on the PSA results, as the difference between the deterministic and PSA results for the base case indicates that conducting deterministic analysis introduces bias into the estimated ICER (non-linearity) [33].

**Table 2:** A summary of the parameters used in the model

*Clinical parameters*		
**Parameter**	**Value**	**Source**
Probability of patients having a transfer from a local hospital to an MTC if:		
They were a true positive (ISS ≥ 16 & tool positive)	26.6%	Newgard et al 2016 [19]
They were a false negative (ISS ≥ 16 & tool negative)	32.5%	
They were a true negative (ISS < 16 & tool negative)	4.3%	
They were a false positive (ISS < 16 & tool positive)	7.4%	
Probability of death within 30 days	Risk equation	TARN [15]
Relative risk of death within 30 days of hospitalisation for patients with an ISS ≥ 16 who were treated at a local hospital	1.25	Newgard et al 2013 [3]
Relative risk of death within 30 days of hospitalisation for patients with an ISS < 16 who were treated at a local hospital	1	Assumption
Probability of death between 30 days post-injury and 1-year post-injury for patients with an ISS ≥ 16	3.6%	Mackenzie et al. 2006 [2]
Relative risk of death between 30 days and 1 year post-hospitalisation for patients with an ISS ≥ 16 who were treated at an local hospital	1.64	
Probability of death between 30 days post-injury and 1-year post-injury for patients with an ISS < 16	1.7%	Davidson et al 2011 [20]
Probability of death after 1 year	Age and gender dependant	ONS [18]
Hazard Ratio for the risk of death if someone has a suspected major trauma case with:		
An ISS of less than 16	1.38	Newgard et al 2016 [14]
An ISS of greater than or equal to 16	5.19	Cameron et al. 2005 [4]
*Utility parameters*		
**Parameter**	**Value**	**Source**
Utility for patients with:		
An ISS of 16 or more	0.65	Ahmed et al [21]
An ISS of 15 or less	0.65	
General population utility		
Constant	0.9508566	Ara and Brazier [22]
Age	− 0.0002587	
Age squared	−0.0000332	
Male (1 = male, 0 = otherwise)	0.0212126	
Calculations		
Age and gender matched general population utility for the Ahmed et al population	0.824	Calculated. Mean age was 61 years and 59.1% of the analysis population was male in Ahmed et al. [21]
Utility multipliers, relative to the utility in the general population, for patients with:		
An ISS of 16 or more	0.789	Calculated
An ISS between 15 and 9	0.789	Calculated
An ISS of under 9	1	We assumed that these patients would have a utility equal to that of the general population
*Cost Parameters*		
**Parameter**	**Value**	**Source**
*Admission costs – base case*		
Transfers between local hospitals and MTCs	£252	Assumed to be one additional ambulance call out. NHS improvement [23]. Currency Code ASS02.
MTC admission, if ISS is 16 or over	£2819	NHS improvement [24]
MTC admission, if ISS is less than 16 and over 8	£1466	
*Treatment of a patient with blunt trauma and an ISS in the range of:*		
ISS ≤ 9	£6198	Christensen et al. [25]
9 < ISS ≤ 16	£8989	
16 < ISS ≤ 25	£14,205	
ISS > 25	£21,173	
*Treatment of a patient with penetrating trauma and an ISS in the range of:*		
ISS ≤ 9	£6501	Christensen et al. [26]
9 < ISS ≤ 15	£6035	
15 < ISS ≤ 24	£9453	
24 < ISS ≤ 34	£12,347	
ISS > 34	£16,438	
*Post discharge costs*		
Cost between discharge and 6 months post treatment	£1766	John Nichol, Personal communication
Relative increase in lifetime treatment costs for patients with an ISS ≥ 16 compared to the general population	1.45	Cameron et al. 2006 [27] Delgado et al 2013 [28]
Relative increase in lifetime treatment costs for patients with an ISS < 16 compared to the general population	1.25	Cameron et al. 2006 [27] Delgado et al 2013 [28]
Yearly costs of NHS treatment	Age and gender dependent	Asaria 2017 [29]

NB – distributions and the standard errors around each parameter are provided in the Additional file 1: Appendix

local hospital – local hospital; *MTC* major trauma centre, *ISS* injury severity score

In the PSA results, a low sensitivity and high specificity tool results in the least number of cases going to an MTC, the lowest cost and the worst outcomes (probability of death, life expectancy and QALYs). Conversely a highly sensitive and low specificity tool results in the most cases going to an MTC, the highest cost, and the best outcomes. Three strategies have ICERs above £20,000 per QALY gained, but below £30,000 per QALY gained (57% sensitivity, 64.2% sensitivity, 87.5% sensitivity). The two remaining strategies, that were not dominated or extendedly dominated, had ICERs that were above the usual upper limit of NICE’s MAICER of £30,000 per QALY gained.

The model results indicate that out of a population of 100,000 patients to whom a major trauma triage tool was applied, 18,448 out of the 100,000 assessed patients would go to an MTC using the most specific tool whereas 97, 860 of the 100,000 assessed patients would go to the MTC using the most sensitive tool. Even when using a very specific triage tool, the majority patients with an ISS ≥ 16 would go to an MTC due to transfers from the local hospitals.

### Scenario analyses

Table 4 summarises the results of the scenario analyses. When the TARN 2015 survival equation is used and all patients in our simulation are treated as having a missing CCI, the results are remarkably similar to the base case analysis as the strategies which are cost-effective at £20,000 and £30,000 per QALY gained are the same [16]. In the scenario analyses in which patients with an ISS between 9 and 15 inclusive receive a benefit from MTC care, the conclusions of the base case are changed. If the benefit that these patients receive is 25% or 50% of the benefit accrued from MTC care by patients with an ISS of 16 or more, then the most cost-effective tool at an MAICER of £30,000 per QALY gained is the most sensitive triage tool. Although when these patients receive 50% of the benefit of MTC care, the ICER for the most sensitive strategy is only £20,306 per QALY gained (see Additional file 1: Appendix) indicating that the NICE’s ICER would only have to be a very small amount over the lower end of their usual range of MAICERs to consider the most sensitive rule to be cost-effective in this scenario. In the scenario where these patients receive 75% of the benefit of MTC care that is accrued by patients with an ISS over 16, then the most sensitive triage tool would be cost-effective at MAICERs of both £20,000 and £30,000 per QALY gained.

**Table 3:** The results of the deterministic base case analyses

Triage Tool	Number of cases sent to the MTC per 100,000 patients	Number of cases sent to the MTC per 8916 patients (ISS ≥ 16)	Number of cases sent to the MTC per 91,084 patients (ISS < 16)	Proportion of patients who died before discharge	Proportion of patients who die between discharge and 1-year post-injury	Mean years lived	Mean discounted QALYs	Mean discounted Costs	ICER
Deterministic									
28.4% Sens, 88.6% Spec	18,912	4600	14,312	4.17%	1.80%	32.07	13.620	£32,574	–
57.0% Sens, 80.0% Spec	28,120	6220	21,900	4.14%	1.78%	32.08	13.624	£32,698	ED
64.2% Sens, 76.1% Spec	31,892	6724	25,168	4.12%	1.78%	32.08	13.625	£32,743	ED
69.8% Sens, 70.1% Spec	37,536	7092	30,444	4.11%	1.77%	32.08	13.626	£32,774	ED
74.6% Sens 65.7% Spec	41,672	7392	34,280	4.10%	1.78%	32.08	13.626	£32,793	ED
87.5% Sens, 62.8% Spec	44,976	8156	36,820	4.09%	1.76%	32.09	13.629	£32,854	£33,026
90.4% Sens, 58.4% Spec	49,100	8364	40,736	4.08%	1.75%	32.09	13.630	£32,889	£39,584
94.8% Sens, 18.7% Spec	83,116	8612	74,504	4.08%	1.75%	32.09	13.630	£32,979	ED
99.8% Sens, 2.5% Spec	97,860	8912	88,948	4.06%	1.74%	32.10	13.633	£33,064	£54,515
Probabilistic (all values are mean values)									
28.4% Sens, 88.6% Spec	18,448	4607	13,841	4.78%	1.78%	32.05	13.580	£33,024	–
57.0% Sens, 80.0% Spec	27,670	6331	21,339	4.72%	1.76%	32.07	13.586	£33,181	£25,039
64.2% Sens, 76.1% Spec	31,505	6763	24,741	4.70%	1.75%	32.07	13.588	£33,223	£27,311
69.8% Sens, 70.1% Spec	37,069	7100	29,969	4.69%	1.75%	32.07	13.589	£33,262	ED
74.6% Sens 65.7% Spec	41,192	7388	33,804	4.68%	1.74%	32.08	13.590	£33,294	ED
87.5% Sens, 62.8% Spec	44,499	8165	36,334	4.65%	1.73%	32.08	13.593	£33,363	£27,624
90.4% Sens, 58.4% Spec	48,516	8339	40,177	4.65%	1.73%	32.08	13.594	£33,386	£35,791
94.8% Sens, 18.7% Spec	83,383	8603	74,779	4.64%	1.72%	32.09	13.594	£33,486	ED
99.8% Sens, 2.5% Spec	97,810	8904	88,906	4.62%	1.72%	32.09	13.596	£33,542	£77,477

*MTC* major trauma centre, *ISS* injury severity score, *QALYS* quality adjusted life years, *ICER* incremental cost-effectiveness ratio, *Sens* sensitivity; *Spec* specificity, *ED* extendedly dominated

Table 5 shows the tool that is cost-effective when the cost of MTC care in England is changed. This is assessed at MAICERs of £20,000 and £30,000 per QALY gained. At an MAICER of £20,000 the optimal tool is highly sensitive to the level of best practice tariffs for MTCs in the UK. If the tariffs are set to their 2017/18 levels, then the optimal tool is a highly specific triage tool. If the tariffs were set to £0, then the optimal tool would be a tool with a sensitivity of 88% and a specificity of 63%. These results are similar at MAICERs of £30,000 per QALY gained, with either the tool with a sensitivity of 88% or a sensitivity of 90% being cost-effective.

**Table 4:** The results of the scenario analyses

Scenario	Cost-effective tool at £20,000 per QALY gained	Cost-effective tool at £30,000 per QALY gained
Base Case	28.4% Sens, 88.6% Spec	87.5% Sens, 62.8% Spec
**Scenario analyses**		
TARN 2015 survival equation with every patient’s CCI being missing	28.4% Sens, 88.6% Spec	87.5% Sens, 62.8% Spec
**MTC benefit for people with and ISS in the range 16 > ISS ≥ 9**		
MTCs have 25% benefit RR of death prior to discharge = 1.07 RR of death discharge and one year = 1.16	28.4% Sens, 88.6% Spec	99.8% Sens, 2.5% Spec
MTCs have 50% benefit RR of death prior to discharge = 1.13 RR of death discharge to one year = 1.32	28.4% Sens, 88.6% Spec	99.8% Sens, 2.5% Spec
MTCs have 75% benefit RR of death prior to discharge = 1.19 RR of death discharge to one year = 1.48	99.8% Sens, 2.5% Spec	99.8% Sens, 2.5% Spec

Full results are given in the Additional file 1: Appendix

*QALY* quality adjusted life year, *Sens* sensitivity, *Spec* specificity, *TARN* Trauma Audit and Research Network, *CCI* Charlson comorbidty index, *MTCs* major trauma centres, *RR* relative risk

**Table 5:** Cost-effective triage tool in the threshold analyses on the cost of MTC care in Engalnd

**MAICER = £20,000 per QALY gained**					
*Cost of MTC care for patients with: ISS ≥ 16 (rows) 16 > ISS ≥ 9 (columns)*	*£1541 (2020/21 tariff levels)*	*£1466 (100%)*	*£1099.50 (75%)*	*£733 (50%)*	*£366.50(25%)*
*£2961 (2020/21 tariff levels)*	28.4% Sens 88.6% Spec	28.4% Sens 88.6% Spec	28.4% Sens 88.6% Spec	28.4% Sens 88.6% Spec	57.0% Sens 80.0% Spec
*£2819 (100%)*	28.4% Sens 88.6% Spec	28.4% Sens 88.6% Spec	28.4% Sens 88.6% Spec	28.4% Sens 88.6% Spec	57.0% Sens 80.0% Spec
*£2114.25 (75%)*	28.4% Sens 88.6% Spec	28.4% Sens 88.6% Spec	28.4% Sens 88.6% Spec	28.4% Sens 88.6% Spec	57.0% Sens 80.0% Spec
*£1409.50 (50%)*	28.4% Sens 88.6% Spec	28.4% Sens 88.6% Spec	28.4% Sens 88.6% Spec	57.0% Sens 80.0% Spec	87.5% Sens 62.8% Spec
*£704.75 (25%)*	28.4% Sens 88.6% Spec	28.4% Sens 88.6% Spec	28.4% Sens 88.6% Spec	87.5% Sens 62.8% Spec	90.4% Sens 58.4% Spec
**MAICER = £30,000 per QALY gained**					
*Cost of MTC care for patients with: ISS ≥ 16 (rows) 16 > ISS ≥ 9 (columns)*	*£1541 (2020/21 tariff levels)*	*£1466 (100%)*	*£1099.50 (75%)*	*£733 (50%)*	*£366.50(25%)*
*£1541 (2020/21 tariff levels)*	87.5% Sens 62.8% Spec	87.5% Sens 62.8% Spec	87.5% Sens 62.8% Spec	90.4% Sens 58.4% Spec	87.5% Sens 62.8% Spec
*£2819 (100%)*	87.5% Sens 62.8% Spec	87.5% Sens 62.8% Spec	87.5% Sens 62.8% Spec	87.5% Sens 62.8% Spec	87.5% Sens 62.8% Spec
*£2114.25 (75%)*	87.5% Sens 62.8% Spec	87.5% Sens 62.8% Spec	87.5% Sens 62.8% Spec	90.4% Sens 58.4% Spec	90.4% Sens 58.4% Spec
*£1409.50 (50%)*	90.4% Sens 58.4% Spec	87.5% Sens 62.8% Spec	90.4% Sens 58.4% Spec	90.4% Sens 58.4% Spec	90.4% Sens 58.4% Spec
*£704.75 (25%)*	90.4% Sens 58.4% Spec	90.4% Sens 58.4% Spec	90.4% Sens 58.4% Spec	90.4% Sens 58.4% Spec	99.8% Sens 2.5% Spec

*MAICER* maximum acceptable incremental cost-effectiveness ratio, *ISS* injury severity score, *Sens* sensitivity, *Spec* specificity

Full results as per the base case analysis are available in the Additional file 1: Appendix

## Discussion

### Summary of findings

The cost-effective triage tool for patients with suspected major trauma is highly uncertain, as three potential tools have emerged with ICERs within the range of £20,000 to £30,000 per QALY gained. If the MAICER in the UK for this problem is £20,000 per QALY gained, then the cost-effective triage tool will be a highly specific tool. However, if the MAICER in the UK for this problem is £30,000 per QALY gained then the cost-effective triage tool will be a moderately sensitive tool with a sensitivity in the region of 85 to 90%. The sensitivity of these tools are slightly lower than the American College of Surgeons Committee on Trauma’s (ACSCOT) recommended sensitivity for any new tool of 95% [34]. The key uncertainties in our ICERs relate to the benefit that MTCs offer to patients with an ISS of between 9 and 15. If this subgroup of patients gains a benefit from MTC care, then the cost-effective tool may be a highly sensitive tool. Furthermore, the results are sensitive to the current cost of MTC care in England, which is determined by best practice tariffs payments made to hospitals.

When deciding upon the exact MAICER used for a decision problem in England, NICE committees consider: how certain the ICERs are; whether health related quality of life has been adequately captured in utilities; whether they believe the technology is innovative; and, whether the technology helps the NHS meet its non-health objectives. As the MAICERs increase from £20,000 to £30,000 per QALY gained, the committee will make explicit references to these criteria in their judgement as to whether a new technology is cost-effective. Therefore, if the moderately sensitive tool was to be judged cost-effective in our base case analysis, then the tool would have to be judged as meeting one or more of these additional criteria by a NICE guidelines committee on major trauma.

### Comparison to previous literature

Despite large-scale investment in major trauma networks, the cost-effectiveness of major trauma triage is not well studied, with only one previously published economic model available by Newgard et al [14]. They found in a US setting, implementing a high sensitivity tool (as recommended by ASCOT) was unlikely to be cost-effective. This conclusion is very similar to our findings and shows that developing high sensitivity tools for major trauma are likely to not be a cost-effective use of resources in either the UK or the US based on our current understanding of major trauma systems.

### Strength and limitations

This model is the first model of major trauma set in the UK that we are aware of, follows best practice recommendations for health economic evaluations, and brings together the best available evidence to inform decision making on major trauma centres in the England. However, there are a number of limitations in the underlying evidence base that must be taken into account when considering the results and when designing future research projects designed to make well-informed decisions regarding major trauma care in an English or UK wide context.

Firstly, all the clinical evidence underpinning the model related to a definition of major trauma based solely on patient’s ISS. However, ISS may not be the gold standard definition of major trauma with a new criterion for patients who benefit from major trauma existing [7, 35, 36]. This means that our model cannot reliably estimate the economic benefits of such rules. Secondly, a Dutch cohort was used to simulate patients in our model. This provided a high quality data set which should be representative of patients in the developed world, however complete generalisability to the UK, or other settings cannot be guaranteed [12]. Thirdly, whilst most of the data in this model is from the UK setting, further research on the effectiveness of MTCs, the probability of receiving an secondary transfer to an MTC, the effect of having major trauma on patient’s long-term outcomes in a UK setting, and the total number of patients with an ISS ≥ 16 not transported to an MTC would be desirable. Fourthly, it is thought that patients who go directly to MTCs have better outcomes than patients who receive secondary transfers to the MTC, however quantitative evidence on this effect is lacking meaning that this cannot be accurately quantified in our analyses. Fourthly, the data on the health care costs incurred by major trauma patients in the UK is old, as it uses TARN data from 2000 to 2005. Therefore, an update of this evidence would be a useful addition to this model, particularly if the analysis can present costs associated with care in MTCs and local hospitals. There is a partial update to this evidence, which uses TARN data from 2009 to 2011 [37]. However, the population of this study was limited to patients with major trauma who also had severe bleeding. Finally, to conduct a simulated population it was necessary to exclude patients with missing data from the Dutch Cohort.

### Future research

As highlighted in the strengths and limitations there are several key areas of research that would improve decision making in the area of major trauma triage these include:

1. Estimating the effectiveness of MTC care for patients who meet other criteria of major trauma centre need (e.g. the Lerner et al. criteria), rather than ISS [7]. Generation of this evidence would allow us to update the model so we can reliably estimate the cost-effectiveness of triage tools designed around these criteria.
2. A UK based cohort study should be conducted to assess which patients present to paramedics in a UK setting and which triage rules are effective. This is currently being developed as part of future work packages in the Major Trauma Triage Study.
3. The evidence on the cost of major trauma cases (especially in the long term) in the UK NHS should be updated and differences between the cost of care in MTCs and local hospitals should be explored .
4. Further research should be conducted into whether patients with an ISS of between 9 and 15 receive any benefit from MTC care.
5. Quantifying the benefit of MTCs for patients sent directly to the MTCs and the patients who receive a secondary transfers should estimated separately.

### Implications for practice

This work indicates that based on the current major trauma system in England and the best currently available evidence, if a cost-effective rule is to be selected then we have to accept a non-negligible proportion of severely injured patients (at least 10%) will not initially be identified as needing care at a major trauma centre. Based on current evidence we would expect around 30% of these patients to be transferred to an MTC, but that still leaves 7% of all major trauma cases not receiving the best available care.

## Conclusion

In conclusion, cost-effective prehospital trauma triage involves clinically suboptimal sensitivity unless it can be proved that patients with an ISS between 9 and 15 receive a significant benefit from MTC care. Cost-effective trauma triage tools result in a proportion of severely injured patients (at least 10%) being initially transported to local hospitals. Implementing high sensitivity trauma triage tools in England requires development of more accurate decision rules; research to establish if patients with an ISS between 9 and 15 benefit from MTCs; changes in the MTC funding system in England; or, inefficient use of health care resources to manage patients with less serious injuries at MTCs.

## Supplementary Information

**Additional file 1.** .

## Data Availability

The data from van Rein et al. [8] was accessed by request from MH. The model code, PSA parameters and patient characteristics are available open access under a MIT licence. These are given at: 10.15131/shef.data.13379036.v2

## Abbreviations

MTCs: Major trauma centres
ISS: Injury Severity Score
QALYs: Quality adjusted life years
TARN: Trauma audit research network
GCS: Glasgow coma Scale
NICE: National Institute for Health and Care Excellence
ICER: Incremental cost-effectiveness ratio
MAICER: Maximum acceptable incremental cost-effectiveness ratio
PSA: Probabilistic sensitivity analysis
CCI: Charlson Comorbidity Index

## References

[1] World Health Orgnaisation. Injuries and Violence The Facts 2014. 2014;20. https://apps.who.int/iris/bitstream/handle/10665/149798/9789241508018_eng.pdf;jsessionid=B8DC599DEC637E1B22284BDF61C611CD?sequence=1. Accessed 18 Nov 2020.

[2] MacKenzie EJ, Rivara FP, Jurkovich GJ, Nathens AB, Frey KP, Egleston BL (2006). A National Evaluation of the effect of trauma-center care on mortality. N Engl J Med.

[3] Newgard CD, Staudenmayer K, Hsia RY, Mann NC, Bulger EM, Holmes JF, Fleischman R, Gorman K, Haukoos J, McConnell KJ (2013). The cost of overtriage: more than one-third of low-risk injured patients were taken to major trauma centers. Health Aff.

[4] Cameron CM, Purdie DM, Kliewer EV, McClure RJ. Long-term mortality following trauma: 10 year follow-up in a population-based sample of injured adults. J Trauma Inj Infect Crit Care. 2005. 16361907

[5] Cudnik MT, Newgard CD, Sayre MR, Steinberg SM (2009). Level i versus level II trauma centers: an outcomes-based assessment. J Trauma Inj Infect Crit Care.

[6] Polites SF, Leonard JM, Glasgow AE, Zielinski MD, Jenkins DH, Habermann EB. Undertriage after severe injury among United States trauma centers and the impact on mortality. Am J Surg. 2018. 10.1016/j.amjsurg.2018.07.061 30241769

[7] Lerner EB, Willenbring BD, Pirrallo RG, Brasel KJ, Cady CE, Colella MR, et al. A consensus-based criterion standard for trauma center need. In: J Trauma Acute Care Surg. 2014. 10.1097/TA.0000000000000189 24662885

[8] van Rein EAJ, van der Sluijs R, Houwert RM, Gunning AC, Lichtveld RA, Leenen LPH, et al. Effectiveness of prehospital trauma triage systems in selecting severely injured patients: is comparative analysis possible? American journal of emergency medicine. 2018. 10.1016/j.ajem.2018.01.055 29395772

[9] National Institute for Health and Care Excellence. Guide to the methods of technology appraisal. Online Source. 2013;1–93. Available Last Accessed: 17 Mar 2016. doi:10.2165/00019053-200826090-00002. 27905712

[10] Newgard CD, Hsia RY, Mann NC, Schmidt T, Sahni R, Bulger EM, et al. The trade-offs in field trauma triage: a multiregion assessment of accuracy metrics and volume shifts associated with different triage strategies. J Trauma Acute Care Surg. 2013. 10.1097/TA.0b013e31828b7848 PMC3726266 23609282

[11] Shanahan T, Fuller GW, Sheldon T, Turton E, Quilty FM, Marincowitz C. External validation of the Dutch prediction model for prehospital triage of trauma patients in South West region of England, United Kingdom. Injury. 2021;52(5):1108–16. 10.1016/j.injury.2021.01.039 33581872

[12] Van Rein EAJ, Van Der Sluijs R, Voskens FJ, KWW L, Houwert RM, Lichtveld RA, et al. Development and Validation of a Prediction Model for Prehospital Triage of Trauma Patients. JAMA Surg. 2019;154(5):421–9. 10.1001/jamasurg.2018.4752 PMC6537785 30725101

[13] Chesser TJ, Moran C, Willett K, Bouillon B, Sturm J, Flohé S (2019). Development of trauma systems in Europe—reports from England, Germany, the Netherlands, and Spain. OTA Int Open Access J Orthop Trauma.

[14] Newgard CD, Yang Z, Nishijima D, KJ MC, Trent SA, Holmes JF, et al. Cost-effectiveness of field trauma triage among injured adults served by emergency medical services. J Am Coll Surg. 2016;222(6):1125-37. 10.1016/j.jamcollsurg.2016.02.014 PMC4975576 27178369

[15] Bouamra O, Wrotchford A, Hollis S, Vail A, Woodford M, Lecky F. Outcome prediction in trauma. Injury. 2006;37(12):1092-7. 10.1016/j.injury.2006.07.029 17087959

[16] Bouamra O, Jacques R, Edwards A, Yates DW, Lawrence T, Jenks T, Woodford M, Lecky F (2015). Prediction modelling for trauma using comorbidity and “true” 30-day outcome. Emerg Med J.

[17] R Core Team (2019). R: A language and environment for statistical computing. 2019. Accessed 1 April 2019.

[18] Office for National Statistics. National life tables: UK 2015-2017. 2021. Online Source Available from https//www.ons.gov.uk/peoplepopulationandcommunity/birthsdeathsandmarriages/lifeexpectancies/datasets/nationallifetablesunitedkingdomreferencetables.

[19] Newgard CD, Yang Z, Nishijima D, McConnell KJ, Trent SA, Holmes JF (2016). Cost-effectiveness of field trauma triage among injured adults served by emergency medical services. J Am Coll Surg.

[20] Davidson GH, Hamlat CA, Rivara FP, Koepsell TD, Jurkovich GJ, Arbabi S. Long-term survival of adult trauma patients. JAMA. 2011. 10.1001/jama.2011.259 21386078

[21] W. A, R. A, Ahmed W, Alwe R, Wade D. One-year functional outcomes following major trauma: experience of a UK level 1 major trauma centre. Clin Rehabil. 2017;31:1646–52. 10.1177/0269215517712044. 10.1177/0269215517712044 28580790

[22] Ara R, Brazier JE (2010). Populating an economic model with health state utility values: moving toward better practice. Value Health.

[23] N18 and /18 Reference cost data. Online source available from https://improvement.nhs.uk/resources/reference-costs/. 2019. Last Accessed 19 July 2019.

[24] N18 and /18 and 2018/19 National Tariff Payment System. Online Source Available from https://improvementnhsuk/documents/1044/2017-18_and_2018-19_National_Tariff_Payment_System pdf. 2019. Last Accessed 19th July 2019.

[25] Christensen MC, Ridley S, Lecky FE, Munro V, Morris S. Outcomes and costs of blunt trauma in England and Wales. Crit Care. 2008. 10.1186/cc6797 PMC2374581 18298813

[26] Christensen MC, Nielsen TG, Ridley S, Lecky FE, Morris S. Outcomes and costs of penetrating trauma injury in England and Wales. Injury. 2008. 10.1016/j.injury.2008.01.012 18417132

[27] Cameron CM, Purdie DM, Kliewer EV, McClure RJ (2006). Ten-year health service use outcomes in a population-based cohort of 21 000 injured adults: the Manitoba injury outcome study. Bull World Health Organ.

[28] Delgado MK, Staudenmayer KL, Wang NE, Spain DA, Weir S, Owens DK, et al. Cost-effectiveness of helicopter versus ground emergency medical services for trauma scene transport in the United States. Ann Emerg Med. 2013;62(4):351–64. 10.1016/j.annemergmed.2013.02.025 PMC3999834 23582619

[29] Asaria M. Health care costs in the English NHS: reference tables for average annual NHS spend by age, sex and deprivation group. Unit Costs Heal Soc Care. 2017:16–21.

[30] National institute for health and care excellence. Position statement on use of the EQ-5D-5L value set for England (updated October 2019). Online Source 2019. https://www.nice.org.uk/about/what-we-do/our-programmes/nice-guidance/technology-appraisal-guidance/eq-5d-5l. Accessed 10 Nov 2021.

[31] Ara R, Brazier JE (2010). Populating an economic model with health state utility values: moving toward better practice. Value Health.

[32] Curtis L, Burns A. Unit Costs of Health and Social Care 2018. Online Source. 2019. Available Last Accessed 19 July 2019.

[33] Hatswell AJ, Bullement A, Briggs A, Paulden M, Stevenson MD (2018). Probabilistic sensitivity analysis in cost-effectiveness models: determining model convergence in cohort models. Pharmacoeconomics..

[34] Criteria R. Resources for optimal care of the injured patient 2006. Chicago Am …. 2006.

[35] Newgard CD, Fu R, Zive D, Rea T, Malveau S, Daya M, et al. Prospective validation of the National Field Triage Guidelines for identifying seriously injured persons. J Am Coll Surg. 2016;222(2):146–158. 10.1016/j.jamcollsurg.2015.10.016. 10.1016/j.jamcollsurg.2015.10.016 PMC5323436 26712244

[36] van der Sluijs R, Lokerman RD, Waalwijk JF, de Jongh MAC, Edwards MJR, den Hartog D, Giannakópoulos GF, van Grunsven PM, Poeze M, Leenen LPH, van Heijl M, Lansink KWW, Breeman W, Bevelander T, Siegers A, van Vliet R, Verhagen TMF, Hoogeveen MWMJ, Sturms LM (2020). Accuracy of pre-hospital trauma triage and field triage decision rules in children (P2-T2 study): an observational study. Lancet Child Adolesc Heal.

[37] Campbell HE, Stokes EA, Bargo DN, Curry N, Lecky FE, Edwards A, Woodford M, Seeney F, Eaglestone S, Brohi K, Gray AM, Stanworth SJ (2015). Quantifying the healthcare costs of treating severely bleeding major trauma patients: a national study for England. Crit Care.

