# Supplementary material for: An economic evaluation of triage tools for patients with suspected severe injuries in England
Source: BMC Emerg Med. 2022 Jan 11;22:4. doi: 10.1186/s12873-021-00557-6 (PMC8753918; doi:10.1186/s12873-021-00557-6)
Supplement: Supplementary file 1 — Additional file 1. . [file 12873_2021_557_MOESM1_ESM.docx]

Contents

[Distributions around each parameter in the PSA 2](#_Toc57722658)

[Details of how to generate the simulated population 7](#_Toc57722659)

[Derivation of the formula to determine QALYs in the model 12](#_Toc57722660)

[Stability of the base case model to the number of patients run through the model 14](#_Toc57722661)

[Details on how the use of MTCs was adjusted for in the TARN 2015 survival equation 18](#_Toc57722662)

[Full results for the scenario analyses 19](#_Toc57722663)

[Results of the threshold analyses on best practice tariff payment levels 23](#_Toc57722664)

[References 24](#_Toc57722665)

# Distributions around each parameter in the PSA

|  | **Mean /Alpha** | **Standard Error /Beta** | **Distribution** | **Source** |
| --- | --- | --- | --- | --- |
| **Clinical** | | | | |
| **TARN survival model - 2006** | | | | |
| Age 0 to 5 | 0.328 | 0.238976 | Normal | Bouamra et al 2006.(1) |
| Age 6 to 10 | 0.594 | 0.22261 | Normal | Bouamra et al 2006.(1) |
| Age 11 to 15 | 0.582 | 0.167769 | Normal | Bouamra et al 2006.(1) |
| Age 45 to 54 | -0.335 | 0.116522 | Normal | Bouamra et al 2006.(1) |
| Age 55 to 64 | -0.972 | 0.119901 | Normal | Bouamra et al 2006.(1) |
| Age 65 to 75 | -1.925 | 0.103437 | Normal | Bouamra et al 2006.(1) |
| Age over 75 | -3.167 | 0.130315 | Normal | Bouamra et al 2006.(1) |
| GCS 9 to 12 | -1.089 | 0.097289 | Normal | Bouamra et al 2006.(1) |
| GCS 6 to 8 | -1.533 | 0.108391 | Normal | Bouamra et al 2006.(1) |
| GCS 4 to 5 | -2.689 | 0.119901 | Normal | Bouamra et al 2006.(1) |
| GCS 3 | -3.513 | 0.103437 | Normal | Bouamra et al 2006.(1) |
| GCS intubated | 0 |  | Fixed | Bouamra et al 2006.(1). |
| Square root of (10 / ISS) – 0.953 | -5.46 | 0.319588 | Normal | Bouamra et al 2006.(1) |
| Natural logarithm (ISS/10) – 0.0968 | -4.557 | 0.142762 | Normal | Bouamra et al 2006.(1). |
| Female | -0.213 | 0.699839 | Normal | Bouamra et al 2006.(1) |
| Female & age 0 to 5 | 0.21 | 0.394889 | Normal | Bouamra et al 2006.(1) |
| Female & age 6 to 10 | 0.008 | 0.44676 | Normal | Bouamra et al 2006.(1) |
| Female & age 11 to 15 | 0.143 | 0.307142 | Normal | Bouamra et al 2006.(1). |
| Female & age 45 to 54 | 0.018 | 0.221073 | Normal | Bouamra et al 2006.(1) |
| Female & age 55 to 64 | 0.521 | 0.221773 | Normal | Bouamra et al 2006.(1) |
| Female & age 65 to 75 | 0.623 | 0.187478 | Normal | Bouamra et al 2006.(1). |
| Female & age over 75 | 0.56 | 0.144961 | Normal | Bouamra et al 2006.(1). |
| Constant | 5.294 |  | Fixed | Bouamra et al 2006.(1) |
| **TARN survival model - 2015** | | | | |
| Square root of (10/ISS) – 0.8686 | -2.79052 | 0.142762 | Normal | Bouamra et al. 2015.(2) |
| Natural logarithm of (ISS/10) – 0.2817 | -2.57574 | 0.073486 | Normal | Bouamra et al. 2015.(2) |
| GCS =3 | -3.79637 | 0.051078 | Normal | Bouamra et al. 2015.(2) |
| GCS 4 to 5 | -2.73865 | 0.076236 | Normal | Bouamra et al. 2015.(2) |
| GCS 6 to 8 | -1.87664 | 0.060018 | Normal | Bouamra et al. 2015.(2) |
| GCS 9 to 12 | -1.29443 | 0.05166 | Normal | Bouamra et al. 2015.(2) |
| GCS 13 to 14 | -0.46062 | 0.038295 | Normal | Bouamra et al. 2015.(2) |
| GCS intubated | -2.62397 | 0.100996 | Normal | Bouamra et al. 2015.(2) |
| CCI unknown | -0.449 | 0.038455 | Normal | Bouamra et al. 2015.(2) |
| CCI 1to 5 | -0.49572 | 0.034608 | Normal | Bouamra et al. 2015.(2) |
| CCI 6 to 10 | -0.96308 | 0.04811 | Normal | Bouamra et al. 2015.(2) |
| CCI over 10 | -1.59703 | 0.062588 | Normal | Bouamra et al. 2015.(2) |
| Age 0 to 5 | -0.00483 | 0.164738 | Normal | Bouamra et al. 2015.(2) |
| Age 6 to 10 | 0.25323 | 0.232061 | Normal | Bouamra et al. 2015.(2) |
| Age 11 to 15 | -0.08435 | 0.151872 | Normal | Bouamra et al. 2015.(2) |
| Age 45 to 54 | -0.41388 | 0.067219 | Normal | Bouamra et al. 2015.(2) |
| Age 55 to 64 | -0.93229 | 0.066243 | Normal | Bouamra et al. 2015.(2) |
| Age 65 to 74 | -1.58082 | 0.064409 | Normal | Bouamra et al. 2015.(2) |
| Age over 75 | -2.6752 | 0.053201 | Normal | Bouamra et al. 2015.(2) |
| Female | -0.17252 | 0.078806 | Normal | Bouamra et al. 2015.(2) |
| Female & Age 0 to 5 | -0.13805 | 0.251346 | Normal | Bouamra et al. 2015.(2) |
| Female & Age 6 to 10 | 0.43973 | 0.442748 | Normal | Bouamra et al. 2015.(2) |
| Female & Age 11 to 15 | 0.21675 | 0.295405 | Normal | Bouamra et al. 2015.(2) |
| Female & Age 45 to 54 | -0.06972 | 0.133246 | Normal | Bouamra et al. 2015.(2) |
| Female & Age 55 to 64 | 0.17164 | 0.121855 | Normal | Bouamra et al. 2015.(2) |
| Female & age 65 to 74 | 0.25829 | 0.112944 | Normal | Bouamra et al. 2015.(2) |
| Female & age over 75 | 0.3477 | 0.087463 | Normal | Bouamra et al. 2015.(2) |
| Constant | 5.28621 |  | Fixed | Bouamra et al. 2015.(2) |
| Probability that someone with an ISS of 16 or more went to an MTC in the UK | 31865 | 9438.102 | Beta | Moran, C.G., et al, 2018. (3) |
| Relative risk of death in hospital for patients with an ISS of over 15 in a local hospital compared to an MTC | 0.2231 | 0.1138 | Log Normal | Newgard et al 2016(4)  Newgard et al. 2013. |
| Probability of patients having a transfer from a local hospital to an MTC if they were a true positive | 90 | 248 | Beta | Newgard et al. 2016.(4) |
| Probability of patients having a transfer from a local hospital to an MTC if they were a false negative | 110 | 228 | Beta | Newgard et al. 2016.(4) |
| Probability of patients having a transfer from a local hospital to an MTC if they were a true negative | 393 | 4918 | Beta | Newgard et al. 2016.(4) |
| Probability of patients having a transfer from a local hospital to an MTC if they were a false negative | 228 | 5083 | Beta | Newgard et al. 2016.(4) |
| Probability of death between discharge and one year post injury for patients with an ISS 16 or over who received MTC care | 138 | 4470 | Beta | Mackenzie et al. 2006.(5) |
| Relative Risk of death for people with an ISS 16 or over who received local hospital care (compared to MTC care) | 0.4947 | 0.2131 | Log Normal | Mackenzie et al. 2006.(5) |
| Probability of death between discharge and one-year post injury for patients with an ISS under 16 | 1256 | 72614 | Beta | Davidson et al. 2011(6) JAMA |
| Hazard Ratio of death, more than one year post injury for patients who had an ISS of over 15 compared to the general population | 1.6467 | 0.1406 | Log Normal | Newgard *et al* 2016(4)  Cameron et al. 2006(7) |
| Hazard Ratio of death, more than one year post injury for patients who had an ISS of 15 or under compared to the general population | 0.3221 | 0.1204 | Log Normal | Newgard *et al* 2016(4)  Cameron et al. 2006(7) |
| Utilities | | | | |
| Utility for patients with an ISS of 16 or more | 60.62 | 26.6 | Beta | Ahmed et al.(8) |
| Utility for patients with an ISS of 15 or less | 60.62 | 26.6 | Beta | Ahmed et al.(8) |
| General population utility formula: constant | 0.9508566 | - | Fixed | Ara et al. 2010.(9) |
| General population utility formula: male = 1, 0 = female | 0.0212126 | - | Fixed | Ara et al. 2010.(9) |
| General population utility formula: age | -0.0002587 | - | Fixed | Ara et al. 2010.(9) |
| General population utility formula: age^2 | -0.0000332 | - | Fixed | Ara et al. 2010.(9) |
| Costs | | | | |
| Cost of MTC care if a patient has an ISS between 9 and 15 | 1466 |  | Fixed | NHS improvement.(10) |
| Cost of MTC care if a patient has an ISS over 16 | 2819 |  | Fixed | NHS improvement.(10) |
| Cost of blunt trauma, if a patient’s ISS is 9 or less | 14679.39 | 0.42 | Gamma | Christensen et al 2008. (11) |
| Cost of blunt trauma, if a patient’s ISS is between 10 and 16 | 3141.14 | 2.86 | Gamma | Christensen et al 2008. (11) |
| Cost of blunt trauma, if a patient’s ISS is between 17 and 25 | 2641.31 | 5.38 | Gamma | Christensen et al 2008. (11) |
| Cost of blunt trauma, if a patient’s ISS is 26 or more | 2948.91 | 7.18 | Gamma | Christensen et al 2008. (11) |
| Cost of penetrating trauma if a patient’s ISS is 8 or less | 43.90 | 148.08 | Gamma | Christensen et al 2008.(12) |
| Cost of penetrating trauma if a patient’s ISS is between 9 and 15 | 116.47 | 51.81 | Gamma | Christensen et al 2008.(12) |
| Cost of penetrating trauma if a patient’s ISS is between 16 and 24 | 45.03 | 209.93 | Gamma | Christensen et al 2008.(12) |
| Cost of penetrating trauma if a patient’s ISS is between 25 and 34 | 23.14 | 533.64 | Gamma | Christensen et al 2008.(12) |
| Cost of penetrating trauma if a patient’s ISS is 35 or more | 7.08 | 2322.05 | Gamma | Christensen et al 2008.(12) |
| Cost of major trauma between discharge and 6 months post-discharge | 1740.76 | 1.01 | Gamma | Personal Communication John Nichol |
| Cost of additional ambulance journeys | 577.14 | 0.44 | Gamma | NHS improvement.(13) Currency Code ASS02. Standard error assumed to be 4.2% of mean, based on analysis of the same currency code in the 2016/17 reference costs. |
| Relative increase in lifetime health care costs for people with an ISS of 16 or more | 1.65 | 0.14 | Log Normal | Cameron et al. 2006(14) |
| Relative increase in lifetime health care costs for people with an ISS of 15 or less | 0.32 | 0.12 | Log Normal | Cameron et al. 2006(14) |

# Details of how to generate the simulated population

Random samples were taken from a multivariate normal distribution. The means and the covariance matrix required to generate the simulated population are given in Table X1.1

Table X1.1: The means and covariance matrix required to populate a multivariate normal distribution

| **Characteristic** | **Mean** | **Covariance matrix** | | | | |
| --- | --- | --- | --- | --- | --- | --- |
|  |  | Age | Percentage Male | ISS | GCS | Percentage with blunt trauma |
| Age | 46.77 | 455.69 | -1.12 | 18.78 | -0.84 | 0.06 |
| Percentage Male | 0.58 | -1.12 | 0.24 | 0.25 | -0.04 | 0.00 |
| ISS | 5.17 | 18.78 | 0.25 | 51.98 | -5.80 | -0.07 |
| GCS | 14.42 | -0.84 | -0.04 | -5.80 | 3.64 | 0.01 |
| Percentage with blunt trauma | 0.98 | 0.06 | 0.00 | -0.07 | 0.01 | 0.02 |
| SD, standard deviation; ISS, injury severity score; GCS, Glasgow coma score | | | | | | |

Samples for the each variable was compared to the following lookup tables. The lookup tables were generated by sorting each variable into a numerically ascending order. The cumulative percentage that each category and preceding categories had out of the sample size was calculated. We then calculated sampling cut-offs, which are the values from a normal distribution defined by percentile = the cumulative percentage, mean = mean value of the variable in Table X1.1 and SD = Variance of variable^0.5 (i.e. for age this would be square root of the cell in covariance matrix in Table X1.1 corresponding to the row and column for Age). The sampled values were then compared sequentially to each sampling cutoff, the simulated value for the variable corresponded to the last category in which the sampled value was less than the sampling cut-off. For example, if a sampled value of 25.5 was drawn for a patient’s age, then they were assigned an age of 22, as 22.5 is less than 25.88 (cut-off for age 22) but more than 23.68 (cut-off for age 21).

Table X1.2: Lookup table for age

| Age | n | cumulative percentage | sampling cut-offs |
| --- | --- | --- | --- |
| 16 | 79 | 0.016737288 | 1.374472 |
| 17 | 106 | 0.039194915 | 9.192516 |
| 18 | 127 | 0.066101695 | 14.6282 |
| 19 | 117 | 0.090889831 | 18.26089 |
| 20 | 105 | 0.113135593 | 20.93512 |
| 21 | 126 | 0.139830508 | 23.68757 |
| 22 | 114 | 0.163983051 | 25.88335 |
| 23 | 103 | 0.185805085 | 27.69266 |
| 24 | 91 | 0.205084746 | 29.18408 |
| 25 | 91 | 0.224364407 | 30.59424 |
| 26 | 86 | 0.242584746 | 31.86488 |
| 27 | 83 | 0.260169492 | 33.043 |
| 28 | 53 | 0.271398305 | 33.77378 |
| 29 | 72 | 0.286652542 | 34.7429 |
| 30 | 74 | 0.302330508 | 35.71378 |
| 31 | 63 | 0.315677966 | 36.52262 |
| 32 | 62 | 0.328813559 | 37.3045 |
| 33 | 59 | 0.341313559 | 38.03693 |
| 34 | 52 | 0.352330508 | 38.67402 |
| 35 | 48 | 0.3625 | 39.25576 |
| 36 | 62 | 0.375635593 | 39.99908 |
| 37 | 66 | 0.389618644 | 40.78145 |
| 38 | 54 | 0.401059322 | 41.4156 |
| 39 | 52 | 0.412076271 | 42.02183 |
| 40 | 63 | 0.425423729 | 42.75125 |
| 41 | 86 | 0.443644068 | 43.73962 |
| 42 | 74 | 0.459322034 | 44.58485 |
| 43 | 77 | 0.475635593 | 45.46074 |
| 44 | 77 | 0.491949153 | 46.33444 |
| 45 | 72 | 0.50720339 | 47.15072 |
| 46 | 64 | 0.520762712 | 47.87674 |
| 47 | 60 | 0.533474576 | 48.55854 |
| 48 | 70 | 0.548305085 | 49.35634 |
| 49 | 67 | 0.5625 | 50.12334 |
| 50 | 69 | 0.577118644 | 50.91781 |
| 51 | 76 | 0.593220339 | 51.79963 |
| 52 | 69 | 0.607838983 | 52.6077 |
| 53 | 73 | 0.623305085 | 53.47186 |
| 54 | 72 | 0.638559322 | 54.33508 |
| 55 | 82 | 0.655932203 | 55.3336 |
| 56 | 66 | 0.669915254 | 56.15102 |
| 57 | 54 | 0.681355932 | 56.83017 |
| 58 | 62 | 0.694491525 | 57.62275 |
| 59 | 56 | 0.706355932 | 58.35169 |
| 60 | 67 | 0.720550847 | 59.24204 |
| 61 | 58 | 0.732838983 | 60.03066 |
| 62 | 67 | 0.747033898 | 60.96486 |
| 63 | 53 | 0.758262712 | 61.72354 |
| 64 | 47 | 0.768220339 | 62.41247 |
| 65 | 54 | 0.779661017 | 63.22473 |
| 66 | 49 | 0.790042373 | 63.98296 |
| 67 | 51 | 0.800847458 | 64.79593 |
| 68 | 52 | 0.811864407 | 65.65269 |
| 69 | 50 | 0.822457627 | 66.50622 |
| 70 | 41 | 0.831144068 | 67.23041 |
| 71 | 54 | 0.842584746 | 68.22179 |
| 72 | 47 | 0.852542373 | 69.12395 |
| 73 | 43 | 0.861652542 | 69.98581 |
| 74 | 39 | 0.869915254 | 70.80162 |
| 75 | 46 | 0.879661017 | 71.8114 |
| 76 | 41 | 0.888347458 | 72.76118 |
| 77 | 41 | 0.897033898 | 73.76541 |
| 78 | 35 | 0.904449153 | 74.67258 |
| 79 | 41 | 0.913135593 | 75.80381 |
| 80 | 30 | 0.919491525 | 76.68628 |
| 81 | 43 | 0.928601695 | 78.04813 |
| 82 | 36 | 0.936228814 | 79.29499 |
| 83 | 37 | 0.944067797 | 80.70396 |
| 84 | 31 | 0.950635593 | 82.00994 |
| 85 | 22 | 0.95529661 | 83.02365 |
| 86 | 39 | 0.963559322 | 85.05241 |
| 87 | 23 | 0.968432203 | 86.43275 |
| 88 | 35 | 0.975847458 | 88.91836 |
| 89 | 15 | 0.979025424 | 90.18529 |
| 90 | 21 | 0.983474576 | 92.26534 |
| 91 | 12 | 0.986016949 | 93.68056 |
| 92 | 17 | 0.989618644 | 96.125 |
| 93 | 13 | 0.992372881 | 98.55885 |
| 94 | 8 | 0.994067797 | 100.4772 |
| 95 | 14 | 0.997033898 | 105.5013 |
| 96.5* | 7 | 0.998516949 | 110.1917 |
| 99* | 7 | 1 | Inf |
| * - more than one age band for identifiability reasons, as at least one category had n < 5. | | | |

Table X1.3: The lookup table for Male gender

| Male = 1, Female = 0 | n | cumulative percentage | sampling cut-offs |
| --- | --- | --- | --- |
| 0 | 1969 | 0.417161 | 0.479693 |
| 1 | 2751 | 1 | Inf |

Table X1.4: The lookup table for ISS

| ISS | n | cumulative percentage | sampling cut-offs |
| --- | --- | --- | --- |
| 0 | 302 | 0.063983 | -5.80918 |
| 1 | 1761 | 0.437076 | 4.023145 |
| 2 | 619 | 0.56822 | 6.403973 |
| 3 | 81 | 0.585381 | 6.720004 |
| 4 | 493 | 0.689831 | 8.736452 |
| 5 | 226 | 0.737712 | 9.75255 |
| 6 | 68 | 0.752119 | 10.07601 |
| 8 | 38 | 0.760169 | 10.26112 |
| 9 | 378 | 0.840254 | 12.3422 |
| 10 | 183 | 0.879025 | 13.60118 |
| 11 | 39 | 0.887288 | 13.90472 |
| 12 | 12 | 0.889831 | 14.0013 |
| 13 | 39 | 0.898093 | 14.3267 |
| 14 | 53 | 0.909322 | 14.80131 |
| 16 | 50 | 0.919915 | 15.29091 |
| 17 | 73 | 0.935381 | 16.10282 |
| 18 | 19 | 0.939407 | 16.33854 |
| 19 | 14 | 0.942373 | 16.5202 |
| 20 | 33 | 0.949364 | 16.97954 |
| 21 | 36 | 0.956992 | 17.5424 |
| 22 | 22 | 0.961653 | 17.9274 |
| 24 | 12 | 0.964195 | 18.15374 |
| 25 | 27 | 0.969915 | 18.71581 |
| 26 | 23 | 0.974788 | 19.26952 |
| 27 | 11 | 0.977119 | 19.56672 |
| 29 | 29 | 0.983263 | 20.4951 |
| 30 | 6 | 0.984534 | 20.72291 |
| 32 | 8 | 0.986229 | 21.05311 |
| 33 | 6 | 0.9875 | 21.32462 |
| 34.5* | 19 | 0.991525 | 22.38014 |
| 37* | 8 | 0.99322 | 22.96356 |
| 41.5* | 9 | 0.995127 | 23.7998 |
| 43 | 5 | 0.996186 | 24.40125 |
| 45 | 10 | 0.998305 | 26.28897 |
| 51.67* | 8 | 1 | Inf |
| * - more than one ISS category has been combined, as at least one of the merged categories had n < 5 | | | |

Table X1.5: The lookup table for GCS

| GCS | n | cumulative percentage | sampling cut-offs |
| --- | --- | --- | --- |
| 3 | 65 | 0.013771 | 10.21646 |
| 4 | 10 | 0.01589 | 10.32435 |
| 5 | 7 | 0.017373 | 10.39275 |
| 6 | 21 | 0.021822 | 10.57168 |
| 7 | 24 | 0.026907 | 10.74172 |
| 8 | 14 | 0.029873 | 10.82882 |
| 9 | 20 | 0.03411 | 10.94157 |
| 10 | 32 | 0.04089 | 11.10006 |
| 11 | 33 | 0.047881 | 11.24253 |
| 12 | 58 | 0.060169 | 11.4569 |
| 13 | 139 | 0.089619 | 11.85788 |
| 14 | 437 | 0.182203 | 12.68964 |
| 15 | 3860 | 1 | Inf |

Table X1.6: The lookup table for blunt trauma

| Blunt trauma = 1, penetrating trauma = 0 | n | cumulative percentage | sampling cut-offs |
| --- | --- | --- | --- |
| 0 | 83 | 0.017585 | 0.705528 |
| 1 | 4637 | 1 | Inf |

# Derivation of the formula to determine QALYs in the model

This section details how the formula used in our model to determine undiscounted and discounted quality adjusted life years (QALYs) was derived.

*Undiscounted QALYs*

For each patient, their age-adjusted utility at any given point in time would be given by the following formula

1. Util now = β1 + β2*(1=Male, 0 = otherwise) + β3*age + β4*age^2

For any given individual patient, this is equivalent to:

1. Util now = β1 + β3*age + β4*age^2

As patient’s gender does not change in our model

Currently age can be expressed as

1. Age = age at baseline + time spent in the model

Substituting 3) into 2) you get:

1. Util now = β1 + β3*(age at baseline + time spent in the model) + β4*( age at baseline + time spent in the model )^2

To get QALYs, we integrate this value with respect to time

For ease of notation: age at baseline = a, time spent in the model = t

$$\int_{-\infty}^{\infty} \beta_{1} + \beta_{3}(a+t) + \beta_{4}\left( a+t \right)^{2}dt$$

Which gives

[β_1_t+ 0.5t^2^(β_3_+2aβ_4_)+at(β_3_+aβ_4_)+( β_4_t^3^)/3] + C

To get QALYs for the general population, you evaluate this formula between age at baseline and age at death

To get QALYs for your patient, you apply utility multipliers to the QALYs for the general population

*Discounted QALYs*

In line with Tappenden *et al*.(15) the standard discounting formula of:

1/(1+dr)^t^

Where dr is the discount rate and t is time since model entry

Can be rewritten as all of the following:

(1+dr)^-t^ & $e^{{ln(1+dr)}^{-t}}$ & $e^{-\ln\left( 1+dr \right)t}$

Consequently the discounted utility of a patient at any point in time is given by the formula:

1. Discounted util now = (β1 + β_3_*(a+t) + β_4_*(a+t)^2^)*$e^{-\ln\left( 1+dr \right)t}$

If we denote -ln(1+dr) = r,

we can do the following integration:

$$\int_{-\infty}^{\infty} \left( \beta_{1} + \beta_{3}\left( a + t \right)+ \beta_{4}\left( a +t \right)^{2} \right)*e^{-rt}dt$$

Which gives:

$$\frac{\boldsymbol{e}^{\boldsymbol{-rt}}\boldsymbol{(-}\beta_{4}\left( a^{2}r^{2}+2ar\left( rt+1 \right)+ r^{2}t^{2}+2rt+2 \right)-r\left( ar\beta_{3}+\beta_{1}r+ \beta_{3}rt+\beta_{3} \right))}{\boldsymbol{r}^{\boldsymbol{3}}}\boldsymbol{+}constant$$

Again, to get lifetime discounted QALYs for someone in the general population who lived as long as your patient, you assess the definite integral of this formula between age at baseline and age at death.

To get discounted QALYs for your patient, you apply utility multipliers to the discounted QALYs for someone in the general population who lived as long as your patient

# Stability of the base case model to the number of patients run through the model

| 1000 patients | | | | | |
| --- | --- | --- | --- | --- | --- |
|  | QALYs | Costs | Incr QALYs | Incr Costs | ICER |
| Sens 28 | 13.844 | £33,945 | - | - | - |
| Sens 57 | 13.844 | £34,004 | - | - | Dominated |
| Sens 64 | 13.844 | £34,021 | - | - | Dominated |
| Sens 70 | 13.849 | £34,111 | 0.005 | £166 | £33,872 |
| Sens 75 | 13.849 | £34,125 | - | - | Dominated |
| Sens 88 | 13.849 | £34,156 | - | - | Dominated |
| Sens 90 | 13.849 | £34,167 | - | - | Dominated |
| Sens 95 | 13.849 | £34,238 | - | - | Dominated |
| Sens 100 | 13.853 | £35,012 | 0.004 | £901 | £215,306 |

| 5000 patients | | | | | |
| --- | --- | --- | --- | --- | --- |
|  | QALYs | Costs | Incr QALYs | Incr Costs | ICER |
| Sens 28 | 13.771 | £34,596 | - | - | - |
| Sens 57 | 13.773 | £34,713 | - | - | ED |
| Sens 64 | 13.773 | £34,737 | - | - | Dominated |
| Sens 70 | 13.776 | £34,780 | 0.006 | £183 | £33,329 |
| Sens 75 | 13.776 | £34,797 | - | - | Dominated |
| Sens 88 | 13.776 | £34,823 | - | - | Dominated |
| Sens 90 | 13.776 | £34,836 | - | - | Dominated |
| Sens 95 | 13.776 | £34,924 | - | - | Dominated |
| Sens 100 | 13.778 | £35,012 | 0.002 | £233 | £122,346 |
| 10000 patients | | | | | |
|  | QALYs | Costs | Incr QALYs | Incr Costs | ICER |
| Sens 28 | 13.707 | £34,475 | - | - | - |
| Sens 57 | 13.709 | £34,588 | - | - | ED |
| Sens 64 | 13.710 | £34,622 | - | - | ED |
| Sens 70 | 13.712 | £34,654 | - | - | ED |
| Sens 75 | 13.713 | £34,690 | 0.006 | £215 | £34,911 |
| Sens 88 | 13.714 | £34,733 | 0.001 | £43 | £44,669 |
| Sens 90 | 13.714 | £34,751 | - | - | ED |
| Sens 95 | 13.714 | £34,836 | - | - | Dominated |
| Sens 100 | 13.715 | £34,903 | 0.001 | £171 | £137,407 |

| 15000 patients | | | | | |
| --- | --- | --- | --- | --- | --- |
|  | QALYs | Costs | Incr QALYs | Incr Costs | ICER |
| Sens 28 | 13.646 | £34,520 | - | - | - |
| Sens 57 | 13.649 | £34,644 | - | - | ED |
| Sens 64 | 13.651 | £34,686 | - | - | ED |
| Sens 70 | 13.652 | £34,714 | - | - | ED |
| Sens 75 | 13.653 | £34,745 | - | - | ED |
| Sens 88 | 13.655 | £34,807 | 0.009 | £287 | £32,520 |
| Sens 90 | 13.655 | £34,824 | - | - | ED |
| Sens 95 | 13.655 | £34,911 | - | - | Dominated |
| Sens 100 | 13.656 | £34,969 | 0.001 | £162 | £187,097 |

| 20000 patients |  |  |  |  |  |
| --- | --- | --- | --- | --- | --- |
|  | QALYs | Costs | Incr QALYs | Incr Costs | ICER |
| Sens 28 | 13.644 | £34,109 | - | - | - |
| Sens 57 | 13.647 | £34,244 | - | - | ED |
| Sens 64 | 13.650 | £34,298 | - | - | ED |
| Sens 70 | 13.651 | £34,325 | - | - | ED |
| Sens 75 | 13.651 | £34,351 | - | - | ED |
| Sens 88 | 13.654 | £34,414 | 0.010 | £305 | £31,792 |
| Sens 90 | 13.654 | £34,430 | - | - | ED |
| Sens 95 | 13.654 | £34,519 | - | - | Dominated |
| Sens 100 | 13.655 | £34,585 | 0.001 | £171 | £135,654 |

| 25000 pats | | | | | |
| --- | --- | --- | --- | --- | --- |
|  | QALYs | Costs | Incr QALYs | Incr Costs | ICER |
| Sens 28 | 13.639 | £34,147 | - | - | - |
| Sens 57 | 13.641 | £34,271 | - | - | ED |
| Sens 64 | 13.644 | £34,320 | - | - | ED |
| Sens 70 | 13.644 | £34,349 | - | - | ED |
| Sens 75 | 13.645 | £34,378 | - | - | ED |
| Sens 88 | 13.648 | £34,441 | - | - | ED |
| Sens 90 | 13.649 | £34,476 | 0.010 | £328 | £31,419 |
| Sens 95 | 13.649 | £34,568 | - | - | ED |
| Sens 100 | 13.651 | £34,644 | 0.002 | £169 | £80,747 |

| 30000 pats | | | | | |
| --- | --- | --- | --- | --- | --- |
|  | QALYs | Costs | Incr QALYs | Incr Costs | ICER |
| Sens 28 | 13.646 | £34,067 | - | - | - |
| Sens 57 | 13.648 | £34,191 | - | - | ED |
| Sens 64 | 13.650 | £34,239 | - | - | ED |
| Sens 70 | 13.651 | £34,267 | - | - | ED |
| Sens 75 | 13.652 | £34,294 | - | - | ED |
| Sens 88 | 13.654 | £34,356 | - | - | ED |
| Sens 90 | 13.655 | £34,389 | 0.010 | £322 | £33,729 |
| Sens 95 | 13.655 | £34,482 | - | - | ED |
| Sens 100 | 13.657 | £34,550 | 0.002 | £161 | £92,590 |

| 40000 pats | | | | | |
| --- | --- | --- | --- | --- | --- |
|  | QALYs | Costs | Incr QALYs | Incr Costs | ICER |
| Sens 28 | 13.666 | £34,133 | - | - | - |
| Sens 57 | 13.669 | £34,254 | - | - | ED |
| Sens 64 | 13.671 | £34,304 | - | - | ED |
| Sens 70 | 13.671 | £34,332 | - | - | ED |
| Sens 75 | 13.672 | £34,357 | - | - | ED |
| Sens 88 | 13.676 | £34,435 | - | - | ED |
| Sens 90 | 13.677 | £34,469 | 0.010 | £336 | £32,392 |
| Sens 95 | 13.677 | £34,562 | - | - | ED |
| Sens 100 | 13.678 | £34,625 | 0.002 | £156 | £96,955 |

| 50000 pats | | | | | |
| --- | --- | --- | --- | --- | --- |
|  | QALYs | Costs | Incr QALYs | Incr Costs | ICER |
| Sens 28 | 13.671 | £34,113 | - | - | - |
| Sens 57 | 13.674 | £34,236 | - | - | ED |
| Sens 64 | 13.676 | £34,283 | - | - | ED |
| Sens 70 | 13.676 | £34,308 | - | - | ED |
| Sens 75 | 13.677 | £34,336 | - | - | ED |
| Sens 88 | 13.681 | £34,422 | - | - | ED |
| Sens 90 | 13.682 | £34,453 | 0.010 | £339 | £32,937 |
| Sens 95 | 13.682 | £34,548 | - | - | ED |
| Sens 100 | 13.683 | £34,610 | 0.002 | £158 | £88,712 |

# Details on how the use of MTCs was adjusted for in the TARN 2015 survival equation

Box 1: Details on how to calculate the risk of death at an MTC and local hospital using the Bouamra *et al* 2015 TARN survival equation.(2)

Mathematical formulae to calculate the TARN risk of death in those patients who did and did not go to an MTC

Where:

p_death_TARN is the probability of death predicted by calculating 1 minus the value of the TARN survival equation

pMTC is the probability that a patient was sent to an MTC

p_death_TARN_MTC is the probability of death for patients in TARN who were sent to the MTC

p_death_TARN_local hospital is the probability of death for patients in TARN who were not sent to the MTC

RR_local hospital_v_MTC is the relative risk of death for patients who are not sent to the MTC compared to those patients who were not

By definition:

1) p_death_TARN = pMTC*p_death_TARN_MTC + (1-pMTC)*p_death_TARN_local hospital

2) p_death_TARN_local hospital = p_death_TARN_MTC*RR_local hospital_v_MTC

Substitute 2) into 1)

3) p_death_TARN = pMTC*p_death_TARN_MTC + (1-pMTC)*(p_death_TARN_MTC*RR_local hospital_v_MTC)

rearrange

4) p_death_TARN = p_death_TARN_MTC*(pMTC+(1-pMTC)* RR_local hospital_v_MTC)

5) p_death_TARN_MTC = p_death_TARN / (pMTC+(1-pMTC)* RR_local hospital_v_MTC)

# Full results for the scenario analyses

**TARN 2015 survival equation with every patient’s CCI being missing**

| **Strategy** | **Probability of been sent to the MTC** | **Probability of been sent to the MTC (ISS ≥ 16)** | **Probability of been sent to the MTC (ISS < 16)** | **Proportion of patients who died before discharge** | **Proportion of patients who die between discharge and 1-year post-injury** | **Mean years lived** | **Mean discounted QALYs** | **Mean discounted Costs** | **ICER** |
| --- | --- | --- | --- | --- | --- | --- | --- | --- | --- |
| **28.4% Sens, 88.6% Spec** | 18.4% | 51.7% | 15.2% | 3.06% | 1.82% | 32.373 | 13.730 | £33,519 | - |
| **57.0% Sens, 80.0% Spec** | 27.7% | 71.0% | 23.4% | 3.00% | 1.79% | 32.386 | 13.736 | £33,667 | £25,427 |
| **64.2% Sens, 76.1% Spec** | 31.5% | 75.9% | 27.2% | 2.99% | 1.78% | 32.389 | 13.738 | £33,708 | £27,601 |
| **69.8% Sens, 70.1% Spec** | 37.1% | 79.6% | 32.9% | 2.98% | 1.78% | 32.392 | 13.739 | £33,746 | ED |
| **74.6% Sens, 65.7% Spec** | 41.2% | 82.9% | 37.1% | 2.97% | 1.77% | 32.394 | 13.740 | £33,777 | ED |
| **87.5% Sens, 62.8% Spec** | 44.5% | 91.6% | 39.9% | 2.95% | 1.76% | 32.400 | 13.742 | £33,841 | £28,146 |
| **90.4% Sens, 58.4% Spec** | 48.5% | 93.5% | 44.1% | 2.94% | 1.76% | 32.401 | 13.743 | £33,864 | £37,007 |
| **94.8% Sens, 18.7% Spec** | 83.4% | 96.5% | 82.1% | 2.94% | 1.75% | 32.403 | 13.744 | £33,962 | ED |
| **99.8% Sens, 2.5% Spec** | 97.8% | 99.9% | 97.6% | 2.93% | 1.75% | 32.405 | 13.745 | £34,017 | £54,102 |

**MTCs have 25% benefit, RR of death prior to discharge = 1.07, RR of death discharge and one year = 1.16**

| **Strategy** | **Probability of been sent to the MTC** | **Probability of been sent to the MTC (ISS ≥ 16)** | **Probability of been sent to the MTC (ISS < 16)** | **Proportion of patients who died before discharge** | **Proportion of patients who die between discharge and 1-year post-injury** | **Mean years lived** | **Mean discounted QALYs** | **Mean discounted Costs** | **ICER** |
| --- | --- | --- | --- | --- | --- | --- | --- | --- | --- |
| **28.4% Sens, 88.6% Spec** | 18.4% | 51.7% | 15.2% | 4.77% | 1.82% | 32.042 | 13.576 | £33,019 | - |
| **57.0% Sens, 80.0% Spec** | 27.7% | 71.0% | 23.4% | 4.71% | 1.79% | 32.058 | 13.583 | £33,176 | £23,141 |
| **64.2% Sens, 76.1% Spec** | 31.5% | 75.9% | 27.2% | 4.69% | 1.78% | 32.062 | 13.585 | £33,219 | £23,802 |
| **69.8% Sens, 70.1% Spec** | 37.1% | 79.6% | 32.9% | 4.68% | 1.77% | 32.066 | 13.586 | £33,259 | ED |
| **74.6% Sens, 65.7% Spec** | 41.2% | 82.9% | 37.1% | 4.67% | 1.77% | 32.069 | 13.588 | £33,292 | ED |
| **87.5% Sens, 62.8% Spec** | 44.5% | 91.6% | 39.9% | 4.64% | 1.75% | 32.076 | 13.591 | £33,361 | £23,853 |
| **90.4% Sens, 58.4% Spec** | 48.5% | 93.5% | 44.1% | 4.63% | 1.75% | 32.078 | 13.592 | £33,384 | £25,355 |
| **94.8% Sens, 18.7% Spec** | 83.4% | 96.5% | 82.1% | 4.61% | 1.73% | 32.087 | 13.595 | £33,489 | ED |
| **99.8% Sens, 2.5% Spec** | 97.8% | 99.9% | 97.6% | 4.60% | 1.72% | 32.092 | 13.597 | £33,547 | £28,930 |

**MTCs have 50% benefit, RR of death prior to discharge = 1.13, RR of death discharge to one year = 1.32**

| **Strategy** | **Probability of been sent to the MTC** | **Probability of been sent to the MTC (ISS ≥ 16)** | **Probability of been sent to the MTC (ISS < 16)** | **Proportion of patients who died before discharge** | **Proportion of patients who die between discharge and 1-year post-injury** | **Mean years lived** | **Mean discounted QALYs** | **Mean discounted Costs** | **ICER** |
| --- | --- | --- | --- | --- | --- | --- | --- | --- | --- |
| **28.4% Sens, 88.6% Spec** | 18.4% | 51.7% | 15.2% | 4.77% | 1.85% | 32.032 | 13.572 | £33,013 | - |
| **57.0% Sens, 80.0% Spec** | 27.7% | 71.0% | 23.4% | 4.70% | 1.82% | 32.049 | 13.580 | £33,172 | ED |
| **64.2% Sens, 76.1% Spec** | 31.5% | 75.9% | 27.2% | 4.69% | 1.81% | 32.054 | 13.582 | £33,215 | ED |
| **69.8% Sens, 70.1% Spec** | 37.1% | 79.6% | 32.9% | 4.67% | 1.80% | 32.058 | 13.584 | £33,256 | ED |
| **74.6% Sens, 65.7% Spec** | 41.2% | 82.9% | 37.1% | 4.66% | 1.79% | 32.062 | 13.585 | £33,289 | ED |
| **87.5% Sens, 62.8% Spec** | 44.5% | 91.6% | 39.9% | 4.63% | 1.78% | 32.070 | 13.589 | £33,358 | ED |
| **90.4% Sens, 58.4% Spec** | 48.5% | 93.5% | 44.1% | 4.62% | 1.77% | 32.072 | 13.590 | £33,382 | ED |
| **94.8% Sens, 18.7% Spec** | 83.4% | 96.5% | 82.1% | 4.59% | 1.74% | 32.088 | 13.596 | £33,491 | ED |
| **99.8% Sens, 2.5% Spec** | 97.8% | 99.9% | 97.6% | 4.57% | 1.72% | 32.096 | 13.599 | £33,551 | £20,368 |

**MTCs have 75% benefit, RR of death prior to discharge = 1.19, RR of death discharge to one year = 1.48**

| **Strategy** | **Probability of been sent to the MTC** | **Probability of been sent to the MTC (ISS ≥ 16)** | **Probability of been sent to the MTC (ISS < 16)** | **Proportion of patients who died before discharge** | **Proportion of patients who die between discharge and 1-year post-injury** | **Mean years lived** | **Mean discounted QALYs** | **Mean discounted Costs** | **ICER** |
| --- | --- | --- | --- | --- | --- | --- | --- | --- | --- |
| **28.4% Sens, 88.6% Spec** | 18.4% | 51.7% | 15.2% | 4.77% | 1.89% | 32.021 | 13.569 | £33,008 | - |
| **57.0% Sens, 80.0% Spec** | 27.7% | 71.0% | 23.4% | 4.70% | 1.85% | 32.040 | 13.576 | £33,167 | ED |
| **64.2% Sens, 76.1% Spec** | 31.5% | 75.9% | 27.2% | 4.68% | 1.84% | 32.045 | 13.579 | £33,211 | ED |
| **69.8% Sens, 70.1% Spec** | 37.1% | 79.6% | 32.9% | 4.66% | 1.83% | 32.051 | 13.581 | £33,252 | ED |
| **74.6% Sens, 65.7% Spec** | 41.2% | 82.9% | 37.1% | 4.65% | 1.82% | 32.055 | 13.583 | £33,286 | ED |
| **87.5% Sens, 62.8% Spec** | 44.5% | 91.6% | 39.9% | 4.62% | 1.80% | 32.063 | 13.586 | £33,356 | ED |
| **90.4% Sens, 58.4% Spec** | 48.5% | 93.5% | 44.1% | 4.61% | 1.80% | 32.067 | 13.588 | £33,380 | ED |
| **94.8% Sens, 18.7% Spec** | 83.4% | 96.5% | 82.1% | 4.57% | 1.75% | 32.088 | 13.596 | £33,493 | ED |
| **99.8% Sens, 2.5% Spec** | 97.8% | 99.9% | 97.6% | 4.55% | 1.72% | 32.099 | 13.600 | £33,554 | £17,299 |

# Results of the threshold analyses on best practice tariff payment levels

| **2020/21 BPT levels, ISS 9 to 15 = £1541, ISS 16+ = £2961** | | | | | | | | | |
| --- | --- | --- | --- | --- | --- | --- | --- | --- | --- |
| **Strategy** | **Probability of been sent to the MTC** | **Probability of been sent to the MTC (ISS ≥ 16)** | **Probability of been sent to the MTC (ISS < 16)** | **Proportion of patients who died before discharge** | **Proportion of patients who die between discharge and 1-year post-injury** | **Mean years lived** | **Mean discounted QALYs** | **Mean discounted Costs** | **ICER** |
| **28.4% Sens, 88.6% Spec** | 18.4% | 51.7% | 15.2% | 4.78% | 1.78% | 32.053 | 13.580 | £33,032 |  |
| **57.0% Sens, 80.0% Spec** | 27.7% | 71.0% | 23.4% | 4.72% | 1.76% | 32.067 | 13.586 | £33,192 | £25,575 |
| **64.2% Sens, 76.1% Spec** | 31.5% | 75.9% | 27.2% | 4.70% | 1.75% | 32.070 | 13.588 | £33,236 | £27,971 |
| **69.8% Sens, 70.1% Spec** | 37.1% | 79.6% | 32.9% | 4.69% | 1.75% | 32.073 | 13.589 | £33,276 | ED |
| **74.6% Sens, 65.7% Spec** | 41.2% | 82.9% | 37.1% | 4.68% | 1.74% | 32.075 | 13.590 | £33,309 | ED |
| **87.5% Sens, 62.8% Spec** | 44.5% | 91.6% | 39.9% | 4.65% | 1.73% | 32.082 | 13.593 | £33,379 | £28,294 |
| **90.4% Sens, 58.4% Spec** | 48.5% | 93.5% | 44.1% | 4.65% | 1.73% | 32.083 | 13.594 | £33,403 | £36,919 |
| **94.8% Sens, 18.7% Spec** | 83.4% | 96.5% | 82.1% | 4.64% | 1.72% | 32.085 | 13.594 | £33,507 | ED |
| **99.8% Sens, 2.5% Spec** | 97.8% | 99.9% | 97.6% | 4.62% | 1.72% | 32.088 | 13.596 | £33,565 | £80,823 |

| **ISS 9 to 15 = £1466, ISS 16+ = £2961** | | | | | | | | | |
| --- | --- | --- | --- | --- | --- | --- | --- | --- | --- |
| **Strategy** | **Probability of been sent to the MTC** | **Probability of been sent to the MTC (ISS ≥ 16)** | **Probability of been sent to the MTC (ISS < 16)** | **Proportion of patients who died before discharge** | **Proportion of patients who die between discharge and 1-year post-injury** | **Mean years lived** | **Mean discounted QALYs** | **Mean discounted Costs** | **ICER** |
| **28.4% Sens, 88.6% Spec** | 18.4% | 51.7% | 15.2% | 4.78% | 1.78% | 32.053 | 13.580 | £33,030 | - |
| **57.0% Sens, 80.0% Spec** | 27.7% | 71.0% | 23.4% | 4.72% | 1.76% | 32.067 | 13.586 | £33,190 | £25,431 |
| **64.2% Sens, 76.1% Spec** | 31.5% | 75.9% | 27.2% | 4.70% | 1.75% | 32.070 | 13.588 | £33,233 | £27,701 |
| **69.8% Sens, 70.1% Spec** | 37.1% | 79.6% | 32.9% | 4.69% | 1.75% | 32.073 | 13.589 | £33,272 | ED |
| **74.6% Sens, 65.7% Spec** | 41.2% | 82.9% | 37.1% | 4.68% | 1.74% | 32.075 | 13.590 | £33,305 | ED |
| **87.5% Sens, 62.8% Spec** | 44.5% | 91.6% | 39.9% | 4.65% | 1.73% | 32.082 | 13.593 | £33,375 | £28,016 |
| **90.4% Sens, 58.4% Spec** | 48.5% | 93.5% | 44.1% | 4.65% | 1.73% | 32.083 | 13.594 | £33,398 | £36,183 |
| **94.8% Sens, 18.7% Spec** | 83.4% | 96.5% | 82.1% | 4.64% | 1.72% | 32.085 | 13.594 | £33,498 | ED |
| **99.8% Sens, 2.5% Spec** | 97.8% | 99.9% | 97.6% | 4.62% | 1.72% | 32.088 | 13.596 | £33,554 | £77,877 |

| **ISS 9 to 15 = £1099.50, ISS 16+ = £2961** | | | | | | | | | |
| --- | --- | --- | --- | --- | --- | --- | --- | --- | --- |
| **Strategy** | **Probability of been sent to the MTC** | **Probability of been sent to the MTC (ISS ≥ 16)** | **Probability of been sent to the MTC (ISS < 16)** | **Proportion of patients who died before discharge** | **Proportion of patients who die between discharge and 1-year post-injury** | **Mean years lived** | **Mean discounted QALYs** | **Mean discounted Costs** | **ICER** |
| **28.4% Sens, 88.6% Spec** | 18.4% | 51.7% | 15.2% | 4.78% | 1.78% | 32.053 | 13.580 | £33,022 | - |
| **57.0% Sens, 80.0% Spec** | 27.7% | 71.0% | 23.4% | 4.72% | 1.76% | 32.067 | 13.586 | £33,177 | £24,718 |
| **64.2% Sens, 76.1% Spec** | 31.5% | 75.9% | 27.2% | 4.70% | 1.75% | 32.070 | 13.588 | £33,218 | £26,406 |
| **69.8% Sens, 70.1% Spec** | 37.1% | 79.6% | 32.9% | 4.69% | 1.75% | 32.073 | 13.589 | £33,255 | ED |
| **74.6% Sens, 65.7% Spec** | 41.2% | 82.9% | 37.1% | 4.68% | 1.74% | 32.075 | 13.590 | £33,285 | ED |
| **87.5% Sens, 62.8% Spec** | 44.5% | 91.6% | 39.9% | 4.65% | 1.73% | 32.082 | 13.593 | £33,353 | £26,657 |
| **90.4% Sens, 58.4% Spec** | 48.5% | 93.5% | 44.1% | 4.65% | 1.73% | 32.083 | 13.594 | £33,374 | £32,612 |
| **94.8% Sens, 18.7% Spec** | 83.4% | 96.5% | 82.1% | 4.64% | 1.72% | 32.085 | 13.594 | £33,453 | ED |
| **99.8% Sens, 2.5% Spec** | 97.8% | 99.9% | 97.6% | 4.62% | 1.72% | 32.088 | 13.596 | £33,502 | £63,465 |

| **ISS 9 to 15 = £733, ISS 16+ = £2961** | | | | | | | | | |
| --- | --- | --- | --- | --- | --- | --- | --- | --- | --- |
| **Strategy** | **Probability of been sent to the MTC** | **Probability of been sent to the MTC (ISS ≥ 16)** | **Probability of been sent to the MTC (ISS < 16)** | **Proportion of patients who died before discharge** | **Proportion of patients who die between discharge and 1-year post-injury** | **Mean years lived** | **Mean discounted QALYs** | **Mean discounted Costs** | **ICER** |
| **28.4% Sens, 88.6% Spec** | 18.4% | 51.7% | 15.2% | 4.78% | 1.78% | 32.053 | 13.580 | £33,014 | - |
| **57.0% Sens, 80.0% Spec** | 27.7% | 71.0% | 23.4% | 4.72% | 1.76% | 32.067 | 13.586 | £33,164 | £24,005 |
| **64.2% Sens, 76.1% Spec** | 31.5% | 75.9% | 27.2% | 4.70% | 1.75% | 32.070 | 13.588 | £33,203 | £25,118 |
| **69.8% Sens, 70.1% Spec** | 37.1% | 79.6% | 32.9% | 4.69% | 1.75% | 32.073 | 13.589 | £33,237 | ED |
| **74.6% Sens, 65.7% Spec** | 41.2% | 82.9% | 37.1% | 4.68% | 1.74% | 32.075 | 13.590 | £33,265 | ED |
| **87.5% Sens, 62.8% Spec** | 44.5% | 91.6% | 39.9% | 4.65% | 1.73% | 32.082 | 13.593 | £33,332 | £25,299 |
| **90.4% Sens, 58.4% Spec** | 48.5% | 93.5% | 44.1% | 4.65% | 1.73% | 32.083 | 13.594 | £33,350 | £29,025 |
| **94.8% Sens, 18.7% Spec** | 83.4% | 96.5% | 82.1% | 4.64% | 1.72% | 32.085 | 13.594 | £33,409 | ED |
| **99.8% Sens, 2.5% Spec** | 97.8% | 99.9% | 97.6% | 4.62% | 1.72% | 32.088 | 13.596 | £33,449 | £49,058 |

| **ISS 9 to 15 = £366.50, ISS 16+ = £2961** | | | | | | | | | |
| --- | --- | --- | --- | --- | --- | --- | --- | --- | --- |
| **Strategy** | **Probability of been sent to the MTC** | **Probability of been sent to the MTC (ISS ≥ 16)** | **Probability of been sent to the MTC (ISS < 16)** | **Proportion of patients who died before discharge** | **Proportion of patients who die between discharge and 1-year post-injury** | **Mean years lived** | **Mean discounted QALYs** | **Mean discounted Costs** | **ICER** |
| **28.4% Sens, 88.6% Spec** | 18.4% | 51.7% | 15.2% | 4.78% | 1.78% | 32.053 | 13.580 | £33,006 | - |
| **57.0% Sens, 80.0% Spec** | 27.7% | 71.0% | 23.4% | 4.72% | 1.76% | 32.067 | 13.586 | £33,152 | £23,293 |
| **64.2% Sens, 76.1% Spec** | 31.5% | 75.9% | 27.2% | 4.70% | 1.75% | 32.070 | 13.588 | £33,189 | £23,823 |
| **69.8% Sens, 70.1% Spec** | 37.1% | 79.6% | 32.9% | 4.69% | 1.75% | 32.073 | 13.589 | £33,219 | ED |
| **74.6% Sens, 65.7% Spec** | 41.2% | 82.9% | 37.1% | 4.68% | 1.74% | 32.075 | 13.590 | £33,245 | ED |
| **87.5% Sens, 62.8% Spec** | 44.5% | 91.6% | 39.9% | 4.65% | 1.73% | 32.082 | 13.593 | £33,310 | £23,940 |
| **90.4% Sens, 58.4% Spec** | 48.5% | 93.5% | 44.1% | 4.65% | 1.73% | 32.083 | 13.594 | £33,326 | £25,454 |
| **94.8% Sens, 18.7% Spec** | 83.4% | 96.5% | 82.1% | 4.64% | 1.72% | 32.085 | 13.594 | £33,365 | ED |
| **99.8% Sens, 2.5% Spec** | 97.8% | 99.9% | 97.6% | 4.62% | 1.72% | 32.088 | 13.596 | £33,396 | £34,646 |

| **ISS 9 to 15 = £1541, ISS 16+ = £2819** | | | | | | | | | |
| --- | --- | --- | --- | --- | --- | --- | --- | --- | --- |
| **Strategy** | **Probability of been sent to the MTC** | **Probability of been sent to the MTC (ISS ≥ 16)** | **Probability of been sent to the MTC (ISS < 16)** | **Proportion of patients who died before discharge** | **Proportion of patients who die between discharge and 1-year post-injury** | **Mean years lived** | **Mean discounted QALYs** | **Mean discounted Costs** | **ICER** |
| **28.4% Sens, 88.6% Spec** | 18.4% | 51.7% | 15.2% | 4.78% | 1.78% | 32.053 | 13.580 | £33,026 | - |
| **57.0% Sens, 80.0% Spec** | 27.7% | 71.0% | 23.4% | 4.72% | 1.76% | 32.067 | 13.586 | £33,183 | £25,183 |
| **64.2% Sens, 76.1% Spec** | 31.5% | 75.9% | 27.2% | 4.70% | 1.75% | 32.070 | 13.588 | £33,226 | £27,579 |
| **69.8% Sens, 70.1% Spec** | 37.1% | 79.6% | 32.9% | 4.69% | 1.75% | 32.073 | 13.589 | £33,266 | ED |
| **74.6% Sens, 65.7% Spec** | 41.2% | 82.9% | 37.1% | 4.68% | 1.74% | 32.075 | 13.590 | £33,298 | ED |
| **87.5% Sens, 62.8% Spec** | 44.5% | 91.6% | 39.9% | 4.65% | 1.73% | 32.082 | 13.593 | £33,368 | £27,901 |
| **90.4% Sens, 58.4% Spec** | 48.5% | 93.5% | 44.1% | 4.65% | 1.73% | 32.083 | 13.594 | £33,391 | £36,512 |
| **94.8% Sens, 18.7% Spec** | 83.4% | 96.5% | 82.1% | 4.64% | 1.72% | 32.085 | 13.594 | £33,495 | ED |
| **99.8% Sens, 2.5% Spec** | 97.8% | 99.9% | 97.6% | 4.62% | 1.72% | 32.088 | 13.596 | £33,553 | £80,430 |

| **ISS 9 to 15 = £1460, ISS 16+ = £2819** | | | | | | | | | |
| --- | --- | --- | --- | --- | --- | --- | --- | --- | --- |
| **Strategy** | **Probability of been sent to the MTC** | **Probability of been sent to the MTC (ISS ≥ 16)** | **Probability of been sent to the MTC (ISS < 16)** | **Proportion of patients who died before discharge** | **Proportion of patients who die between discharge and 1-year post-injury** | **Mean years lived** | **Mean discounted QALYs** | **Mean discounted Costs** | **ICER** |
| **28.4% Sens, 88.6% Spec** | 18.4% | 51.7% | 15.2% | 4.78% | 1.78% | 32.053 | 13.580 | £33,024 | - |
| **57.0% Sens, 80.0% Spec** | 27.7% | 71.0% | 23.4% | 4.72% | 1.76% | 32.067 | 13.586 | £33,181 | £25,040 |
| **64.2% Sens, 76.1% Spec** | 31.5% | 75.9% | 27.2% | 4.70% | 1.75% | 32.070 | 13.588 | £33,223 | £27,310 |
| **69.8% Sens, 70.1% Spec** | 37.1% | 79.6% | 32.9% | 4.69% | 1.75% | 32.073 | 13.589 | £33,262 | ED |
| **74.6% Sens, 65.7% Spec** | 41.2% | 82.9% | 37.1% | 4.68% | 1.74% | 32.075 | 13.590 | £33,294 | ED |
| **87.5% Sens, 62.8% Spec** | 44.5% | 91.6% | 39.9% | 4.65% | 1.73% | 32.082 | 13.593 | £33,363 | £27,623 |
| **90.4% Sens, 58.4% Spec** | 48.5% | 93.5% | 44.1% | 4.65% | 1.73% | 32.083 | 13.594 | £33,386 | £35,792 |
| **94.8% Sens, 18.7% Spec** | 83.4% | 96.5% | 82.1% | 4.64% | 1.72% | 32.085 | 13.594 | £33,486 | ED |
| **99.8% Sens, 2.5% Spec** | 97.8% | 99.9% | 97.6% | 4.62% | 1.72% | 32.088 | 13.596 | £33,542 | £77,479 |

| **ISS 9 to 15 = £1099.50, ISS 16+ = £2819** | | | | | | | | | |
| --- | --- | --- | --- | --- | --- | --- | --- | --- | --- |
| **Strategy** | **Probability of been sent to the MTC** | **Probability of been sent to the MTC (ISS ≥ 16)** | **Probability of been sent to the MTC (ISS < 16)** | **Proportion of patients who died before discharge** | **Proportion of patients who die between discharge and 1-year post-injury** | **Mean years lived** | **Mean discounted QALYs** | **Mean discounted Costs** | **ICER** |
| **28.4% Sens, 88.6% Spec** | 18.4% | 51.7% | 15.2% | 4.78% | 1.78% | 32.053 | 13.580 | £33,016 | - |
| **57.0% Sens, 80.0% Spec** | 27.7% | 71.0% | 23.4% | 4.72% | 1.76% | 32.067 | 13.586 | £33,168 | £24,327 |
| **64.2% Sens, 76.1% Spec** | 31.5% | 75.9% | 27.2% | 4.70% | 1.75% | 32.070 | 13.588 | £33,208 | £26,015 |
| **69.8% Sens, 70.1% Spec** | 37.1% | 79.6% | 32.9% | 4.69% | 1.75% | 32.073 | 13.589 | £33,245 | ED |
| **74.6% Sens, 65.7% Spec** | 41.2% | 82.9% | 37.1% | 4.68% | 1.74% | 32.075 | 13.590 | £33,274 | ED |
| **87.5% Sens, 62.8% Spec** | 44.5% | 91.6% | 39.9% | 4.65% | 1.73% | 32.082 | 13.593 | £33,342 | £26,265 |
| **90.4% Sens, 58.4% Spec** | 48.5% | 93.5% | 44.1% | 4.65% | 1.73% | 32.083 | 13.594 | £33,362 | £32,205 |
| **94.8% Sens, 18.7% Spec** | 83.4% | 96.5% | 82.1% | 4.64% | 1.72% | 32.085 | 13.594 | £33,441 | ED |
| **99.8% Sens, 2.5% Spec** | 97.8% | 99.9% | 97.6% | 4.62% | 1.72% | 32.088 | 13.596 | £33,489 | £63,067 |

| **ISS 9 to 15 = £733, ISS 16+ = £2819** | | | | | | | | | |
| --- | --- | --- | --- | --- | --- | --- | --- | --- | --- |
| **Strategy** | **Probability of been sent to the MTC** | **Probability of been sent to the MTC (ISS ≥ 16)** | **Probability of been sent to the MTC (ISS < 16)** | **Proportion of patients who died before discharge** | **Proportion of patients who die between discharge and 1-year post-injury** | **Mean years lived** | **Mean discounted QALYs** | **Mean discounted Costs** | **ICER** |
| **28.4% Sens, 88.6% Spec** | 18.4% | 51.7% | 15.2% | 4.78% | 1.78% | 32.053 | 13.580 | £33,007 | - |
| **57.0% Sens, 80.0% Spec** | 27.7% | 71.0% | 23.4% | 4.72% | 1.76% | 32.067 | 13.586 | £33,155 | £23,615 |
| **64.2% Sens, 76.1% Spec** | 31.5% | 75.9% | 27.2% | 4.70% | 1.75% | 32.070 | 13.588 | £33,194 | £24,714 |
| **69.8% Sens, 70.1% Spec** | 37.1% | 79.6% | 32.9% | 4.69% | 1.75% | 32.073 | 13.589 | £33,227 | ED |
| **74.6% Sens, 65.7% Spec** | 41.2% | 82.9% | 37.1% | 4.68% | 1.74% | 32.075 | 13.590 | £33,254 | ED |
| **87.5% Sens, 62.8% Spec** | 44.5% | 91.6% | 39.9% | 4.65% | 1.73% | 32.082 | 13.593 | £33,320 | £24,908 |
| **90.4% Sens, 58.4% Spec** | 48.5% | 93.5% | 44.1% | 4.65% | 1.73% | 32.083 | 13.594 | £33,338 | £28,633 |
| **94.8% Sens, 18.7% Spec** | 83.4% | 96.5% | 82.1% | 4.64% | 1.72% | 32.085 | 13.594 | £33,397 | ED |
| **99.8% Sens, 2.5% Spec** | 97.8% | 99.9% | 97.6% | 4.62% | 1.72% | 32.088 | 13.596 | £33,436 | £48,655 |

| **ISS 9 to 15 = £366.50, ISS 16+ = £2819** | | | | | | | | | |
| --- | --- | --- | --- | --- | --- | --- | --- | --- | --- |
| **Strategy** | **Probability of been sent to the MTC** | **Probability of been sent to the MTC (ISS ≥ 16)** | **Probability of been sent to the MTC (ISS < 16)** | **Proportion of patients who died before discharge** | **Proportion of patients who die between discharge and 1-year post-injury** | **Mean years lived** | **Mean discounted QALYs** | **Mean discounted Costs** | **ICER** |
| **28.4% Sens, 88.6% Spec** | 18.4% | 51.7% | 15.2% | 4.78% | 1.78% | 32.053 | 13.580 | £32,999 | - |
| **57.0% Sens, 80.0% Spec** | 27.7% | 71.0% | 23.4% | 4.72% | 1.76% | 32.067 | 13.586 | £33,143 | £22,901 |
| **64.2% Sens, 76.1% Spec** | 31.5% | 75.9% | 27.2% | 4.70% | 1.75% | 32.070 | 13.588 | £33,179 | £23,425 |
| **69.8% Sens, 70.1% Spec** | 37.1% | 79.6% | 32.9% | 4.69% | 1.75% | 32.073 | 13.589 | £33,209 | ED |
| **74.6% Sens, 65.7% Spec** | 41.2% | 82.9% | 37.1% | 4.68% | 1.74% | 32.075 | 13.590 | £33,234 | ED |
| **87.5% Sens, 62.8% Spec** | 44.5% | 91.6% | 39.9% | 4.65% | 1.73% | 32.082 | 13.593 | £33,299 | £23,550 |
| **90.4% Sens, 58.4% Spec** | 48.5% | 93.5% | 44.1% | 4.65% | 1.73% | 32.083 | 13.594 | £33,315 | £25,062 |
| **94.8% Sens, 18.7% Spec** | 83.4% | 96.5% | 82.1% | 4.64% | 1.72% | 32.085 | 13.594 | £33,352 | ED |
| **99.8% Sens, 2.5% Spec** | 97.8% | 99.9% | 97.6% | 4.62% | 1.72% | 32.088 | 13.596 | £33,383 | £34,243 |

| **ISS 9 to 15 = £1541, ISS 16+ = £2114.25** | | | | | | | | | |
| --- | --- | --- | --- | --- | --- | --- | --- | --- | --- |
| **Strategy** | **Probability of been sent to the MTC** | **Probability of been sent to the MTC (ISS ≥ 16)** | **Probability of been sent to the MTC (ISS < 16)** | **Proportion of patients who died before discharge** | **Proportion of patients who die between discharge and 1-year post-injury** | **Mean years lived** | **Mean discounted QALYs** | **Mean discounted Costs** | **ICER** |
| **28.4% Sens, 88.6% Spec** | 18.4% | 51.7% | 15.2% | 4.78% | 1.78% | 32.053 | 13.580 | £32,993 | **-** |
| **57.0% Sens, 80.0% Spec** | 27.7% | 71.0% | 23.4% | 4.72% | 1.76% | 32.067 | 13.586 | £33,139 | £23,243 |
| **64.2% Sens, 76.1% Spec** | 31.5% | 75.9% | 27.2% | 4.70% | 1.75% | 32.070 | 13.588 | £33,179 | £25,618 |
| **69.8% Sens, 70.1% Spec** | 37.1% | 79.6% | 32.9% | 4.69% | 1.75% | 32.073 | 13.589 | £33,216 | ED |
| **74.6% Sens, 65.7% Spec** | 41.2% | 82.9% | 37.1% | 4.68% | 1.74% | 32.075 | 13.590 | £33,246 | ED |
| **87.5% Sens, 62.8% Spec** | 44.5% | 91.6% | 39.9% | 4.65% | 1.73% | 32.082 | 13.593 | £33,310 | £25,955 |
| **90.4% Sens, 58.4% Spec** | 48.5% | 93.5% | 44.1% | 4.65% | 1.73% | 32.083 | 13.594 | £33,332 | £34,586 |
| **94.8% Sens, 18.7% Spec** | 83.4% | 96.5% | 82.1% | 4.64% | 1.72% | 32.085 | 13.594 | £33,434 | ED |
| **99.8% Sens, 2.5% Spec** | 97.8% | 99.9% | 97.6% | 4.62% | 1.72% | 32.088 | 13.596 | £33,490 | £78,450 |

| **ISS 9 to 15 = £1466, ISS 16+ = £2114.25** | | | | | | | | | |
| --- | --- | --- | --- | --- | --- | --- | --- | --- | --- |
| **Strategy** | **Probability of been sent to the MTC** | **Probability of been sent to the MTC (ISS ≥ 16)** | **Probability of been sent to the MTC (ISS < 16)** | **Proportion of patients who died before discharge** | **Proportion of patients who die between discharge and 1-year post-injury** | **Mean years lived** | **Mean discounted QALYs** | **Mean discounted Costs** | **ICER** |
| **28.4% Sens, 88.6% Spec** | 18.4% | 51.7% | 15.2% | 4.78% | 1.78% | 32.053 | 13.580 | £32,991 | **-** |
| **57.0% Sens, 80.0% Spec** | 27.7% | 71.0% | 23.4% | 4.72% | 1.76% | 32.067 | 13.586 | £33,136 | £23,096 |
| **64.2% Sens, 76.1% Spec** | 31.5% | 75.9% | 27.2% | 4.70% | 1.75% | 32.070 | 13.588 | £33,176 | £25,355 |
| **69.8% Sens, 70.1% Spec** | 37.1% | 79.6% | 32.9% | 4.69% | 1.75% | 32.073 | 13.589 | £33,212 | ED |
| **74.6% Sens, 65.7% Spec** | 41.2% | 82.9% | 37.1% | 4.68% | 1.74% | 32.075 | 13.590 | £33,242 | ED |
| **87.5% Sens, 62.8% Spec** | 44.5% | 91.6% | 39.9% | 4.65% | 1.73% | 32.082 | 13.593 | £33,306 | £25,677 |
| **90.4% Sens, 58.4% Spec** | 48.5% | 93.5% | 44.1% | 4.65% | 1.73% | 32.083 | 13.594 | £33,327 | £33,865 |
| **94.8% Sens, 18.7% Spec** | 83.4% | 96.5% | 82.1% | 4.64% | 1.72% | 32.085 | 13.594 | £33,425 | ED |
| **99.8% Sens, 2.5% Spec** | 97.8% | 99.9% | 97.6% | 4.62% | 1.72% | 32.088 | 13.596 | £33,479 | £75,494 |

| **ISS 9 to 15 = £1099.50, ISS 16+ = £2114.25** | | | | | | | | | |
| --- | --- | --- | --- | --- | --- | --- | --- | --- | --- |
| **Strategy** | **Probability of been sent to the MTC** | **Probability of been sent to the MTC (ISS ≥ 16)** | **Probability of been sent to the MTC (ISS < 16)** | **Proportion of patients who died before discharge** | **Proportion of patients who die between discharge and 1-year post-injury** | **Mean years lived** | **Mean discounted QALYs** | **Mean discounted Costs** | **ICER** |
| **28.4% Sens, 88.6% Spec** | 18.4% | 51.7% | 15.2% | 4.78% | 1.78% | 32.053 | 13.580 | £32,983 | - |
| **57.0% Sens, 80.0% Spec** | 27.7% | 71.0% | 23.4% | 4.72% | 1.76% | 32.067 | 13.586 | £33,123 | £22,385 |
| **64.2% Sens, 76.1% Spec** | 31.5% | 75.9% | 27.2% | 4.70% | 1.75% | 32.070 | 13.588 | £33,161 | £24,060 |
| **69.8% Sens, 70.1% Spec** | 37.1% | 79.6% | 32.9% | 4.69% | 1.75% | 32.073 | 13.589 | £33,195 | ED |
| **74.6% Sens, 65.7% Spec** | 41.2% | 82.9% | 37.1% | 4.68% | 1.74% | 32.075 | 13.590 | £33,222 | ED |
| **87.5% Sens, 62.8% Spec** | 44.5% | 91.6% | 39.9% | 4.65% | 1.73% | 32.082 | 13.593 | £33,284 | £24,319 |
| **90.4% Sens, 58.4% Spec** | 48.5% | 93.5% | 44.1% | 4.65% | 1.73% | 32.083 | 13.594 | £33,303 | £30,294 |
| **94.8% Sens, 18.7% Spec** | 83.4% | 96.5% | 82.1% | 4.64% | 1.72% | 32.085 | 13.594 | £33,381 | ED |
| **99.8% Sens, 2.5% Spec** | 97.8% | 99.9% | 97.6% | 4.62% | 1.72% | 32.088 | 13.596 | £33,426 | £61,082 |

| **ISS 9 to 15 = £733, ISS 16+ = £2114.25** | | | | | | | | | |
| --- | --- | --- | --- | --- | --- | --- | --- | --- | --- |
| **Strategy** | **Probability of been sent to the MTC** | **Probability of been sent to the MTC (ISS ≥ 16)** | **Probability of been sent to the MTC (ISS < 16)** | **Proportion of patients who died before discharge** | **Proportion of patients who die between discharge and 1-year post-injury** | **Mean years lived** | **Mean discounted QALYs** | **Mean discounted Costs** | **ICER** |
| **28.4% Sens, 88.6% Spec** | 18.4% | 51.7% | 15.2% | 4.78% | 1.78% | 32.053 | 13.580 | £32,975 | - |
| **57.0% Sens, 80.0% Spec** | 27.7% | 71.0% | 23.4% | 4.72% | 1.76% | 32.067 | 13.586 | £33,111 | £21,672 |
| **64.2% Sens, 76.1% Spec** | 31.5% | 75.9% | 27.2% | 4.70% | 1.75% | 32.070 | 13.588 | £33,146 | £22,765 |
| **69.8% Sens, 70.1% Spec** | 37.1% | 79.6% | 32.9% | 4.69% | 1.75% | 32.073 | 13.589 | £33,177 | ED |
| **74.6% Sens, 65.7% Spec** | 41.2% | 82.9% | 37.1% | 4.68% | 1.74% | 32.075 | 13.590 | £33,202 | ED |
| **87.5% Sens, 62.8% Spec** | 44.5% | 91.6% | 39.9% | 4.65% | 1.73% | 32.082 | 13.593 | £33,263 | £22,960 |
| **90.4% Sens, 58.4% Spec** | 48.5% | 93.5% | 44.1% | 4.65% | 1.73% | 32.083 | 13.594 | £33,280 | £26,707 |
| **94.8% Sens, 18.7% Spec** | 83.4% | 96.5% | 82.1% | 4.64% | 1.72% | 32.085 | 13.594 | £33,336 | ED |
| **99.8% Sens, 2.5% Spec** | 97.8% | 99.9% | 97.6% | 4.62% | 1.72% | 32.088 | 13.596 | £33,373 | £46,675 |

| **ISS 9 to 15 = £366.50, ISS 16+ = £2114.25** | | | | | | | | | |
| --- | --- | --- | --- | --- | --- | --- | --- | --- | --- |
| **Strategy** | **Probability of been sent to the MTC** | **Probability of been sent to the MTC (ISS ≥ 16)** | **Probability of been sent to the MTC (ISS < 16)** | **Proportion of patients who died before discharge** | **Proportion of patients who die between discharge and 1-year post-injury** | **Mean years lived** | **Mean discounted QALYs** | **Mean discounted Costs** | **ICER** |
| **28.4% Sens, 88.6% Spec** | 18.4% | 51.7% | 15.2% | 4.78% | 1.78% | 32.053 | 13.580 | £32,967 |  |
| **57.0% Sens, 80.0% Spec** | 27.7% | 71.0% | 23.4% | 4.72% | 1.76% | 32.067 | 13.586 | £33,098 | £20,961 |
| **64.2% Sens, 76.1% Spec** | 31.5% | 75.9% | 27.2% | 4.70% | 1.75% | 32.070 | 13.588 | £33,131 | £21,470 |
| **69.8% Sens, 70.1% Spec** | 37.1% | 79.6% | 32.9% | 4.69% | 1.75% | 32.073 | 13.589 | £33,159 | ED |
| **74.6% Sens, 65.7% Spec** | 41.2% | 82.9% | 37.1% | 4.68% | 1.74% | 32.075 | 13.590 | £33,182 | ED |
| **87.5% Sens, 62.8% Spec** | 44.5% | 91.6% | 39.9% | 4.65% | 1.73% | 32.082 | 13.593 | £33,241 | £21,602 |
| **90.4% Sens, 58.4% Spec** | 48.5% | 93.5% | 44.1% | 4.65% | 1.73% | 32.083 | 13.594 | £33,256 | £23,135 |
| **94.8% Sens, 18.7% Spec** | 83.4% | 96.5% | 82.1% | 4.64% | 1.72% | 32.085 | 13.594 | £33,292 | ED |
| **99.8% Sens, 2.5% Spec** | 97.8% | 99.9% | 97.6% | 4.62% | 1.72% | 32.088 | 13.596 | £33,321 | £32,263 |

| **ISS 9 to 15 = £1541, ISS 16+ = £1409.50** | | | | | | | | | |
| --- | --- | --- | --- | --- | --- | --- | --- | --- | --- |
| **Strategy** | **Probability of been sent to the MTC** | **Probability of been sent to the MTC (ISS ≥ 16)** | **Probability of been sent to the MTC (ISS < 16)** | **Proportion of patients who died before discharge** | **Proportion of patients who die between discharge and 1-year post-injury** | **Mean years lived** | **Mean discounted QALYs** | **Mean discounted Costs** | **ICER** |
| **28.4% Sens, 88.6% Spec** | 18.4% | 51.7% | 15.2% | 4.78% | 1.78% | 32.053 | 13.580 | £32,961 |  |
| **57.0% Sens, 80.0% Spec** | 27.7% | 71.0% | 23.4% | 4.72% | 1.76% | 32.067 | 13.586 | £33,094 | £21,301 |
| **64.2% Sens, 76.1% Spec** | 31.5% | 75.9% | 27.2% | 4.70% | 1.75% | 32.070 | 13.588 | £33,131 | £23,669 |
| **69.8% Sens, 70.1% Spec** | 37.1% | 79.6% | 32.9% | 4.69% | 1.75% | 32.073 | 13.589 | £33,166 | ED |
| **74.6% Sens, 65.7% Spec** | 41.2% | 82.9% | 37.1% | 4.68% | 1.74% | 32.075 | 13.590 | £33,194 | ED |
| **87.5% Sens, 62.8% Spec** | 44.5% | 91.6% | 39.9% | 4.65% | 1.73% | 32.082 | 13.593 | £33,253 | £24,007 |
| **90.4% Sens, 58.4% Spec** | 48.5% | 93.5% | 44.1% | 4.65% | 1.73% | 32.083 | 13.594 | £33,273 | £32,675 |
| **94.8% Sens, 18.7% Spec** | 83.4% | 96.5% | 82.1% | 4.64% | 1.72% | 32.085 | 13.594 | £33,373 | ED |
| **99.8% Sens, 2.5% Spec** | 97.8% | 99.9% | 97.6% | 4.62% | 1.72% | 32.088 | 13.596 | £33,427 | £76,459 |

| **ISS 9 to 15 = £1466, ISS 16+ = £1409.50** | | | | | | | | | |
| --- | --- | --- | --- | --- | --- | --- | --- | --- | --- |
| **Strategy** | **Probability of been sent to the MTC** | **Probability of been sent to the MTC (ISS ≥ 16)** | **Probability of been sent to the MTC (ISS < 16)** | **Proportion of patients who died before discharge** | **Proportion of patients who die between discharge and 1-year post-injury** | **Mean years lived** | **Mean discounted QALYs** | **Mean discounted Costs** | **ICER** |
| **28.4% Sens, 88.6% Spec** | 18.4% | 51.7% | 15.2% | 4.78% | 1.78% | 32.053 | 13.580 | £32,959 |  |
| **57.0% Sens, 80.0% Spec** | 27.7% | 71.0% | 23.4% | 4.72% | 1.76% | 32.067 | 13.586 | £33,091 | £21,154 |
| **64.2% Sens, 76.1% Spec** | 31.5% | 75.9% | 27.2% | 4.70% | 1.75% | 32.070 | 13.588 | £33,128 | £23,406 |
| **69.8% Sens, 70.1% Spec** | 37.1% | 79.6% | 32.9% | 4.69% | 1.75% | 32.073 | 13.589 | £33,162 | ED |
| **74.6% Sens, 65.7% Spec** | 41.2% | 82.9% | 37.1% | 4.68% | 1.74% | 32.075 | 13.590 | £33,190 | ED |
| **87.5% Sens, 62.8% Spec** | 44.5% | 91.6% | 39.9% | 4.65% | 1.73% | 32.082 | 13.593 | £33,248 | £23,729 |
| **90.4% Sens, 58.4% Spec** | 48.5% | 93.5% | 44.1% | 4.65% | 1.73% | 32.083 | 13.594 | £33,269 | £31,938 |
| **94.8% Sens, 18.7% Spec** | 83.4% | 96.5% | 82.1% | 4.64% | 1.72% | 32.085 | 13.594 | £33,364 | ED |
| **99.8% Sens, 2.5% Spec** | 97.8% | 99.9% | 97.6% | 4.62% | 1.72% | 32.088 | 13.596 | £33,416 | £73,513 |

| **ISS 9 to 15 = £1099.50, ISS 16+ = £1409.50** | | | | | | | | | |
| --- | --- | --- | --- | --- | --- | --- | --- | --- | --- |
| **Strategy** | **Probability of been sent to the MTC** | **Probability of been sent to the MTC (ISS ≥ 16)** | **Probability of been sent to the MTC (ISS < 16)** | **Proportion of patients who died before discharge** | **Proportion of patients who die between discharge and 1-year post-injury** | **Mean years lived** | **Mean discounted QALYs** | **Mean discounted Costs** | **ICER** |
| **28.4% Sens, 88.6% Spec** | 18.4% | 51.7% | 15.2% | 4.78% | 1.78% | 32.053 | 13.580 | £32,951 |  |
| **57.0% Sens, 80.0% Spec** | 27.7% | 71.0% | 23.4% | 4.72% | 1.76% | 32.067 | 13.586 | £33,079 | £20,443 |
| **64.2% Sens, 76.1% Spec** | 31.5% | 75.9% | 27.2% | 4.70% | 1.75% | 32.070 | 13.588 | £33,113 | £22,111 |
| **69.8% Sens, 70.1% Spec** | 37.1% | 79.6% | 32.9% | 4.69% | 1.75% | 32.073 | 13.589 | £33,145 | ED |
| **74.6% Sens, 65.7% Spec** | 41.2% | 82.9% | 37.1% | 4.68% | 1.74% | 32.075 | 13.590 | £33,170 | ED |
| **87.5% Sens, 62.8% Spec** | 44.5% | 91.6% | 39.9% | 4.65% | 1.73% | 32.082 | 13.593 | £33,227 | £22,371 |
| **90.4% Sens, 58.4% Spec** | 48.5% | 93.5% | 44.1% | 4.65% | 1.73% | 32.083 | 13.594 | £33,245 | £28,367 |
| **94.8% Sens, 18.7% Spec** | 83.4% | 96.5% | 82.1% | 4.64% | 1.72% | 32.085 | 13.594 | £33,320 | ED |
| **99.8% Sens, 2.5% Spec** | 97.8% | 99.9% | 97.6% | 4.62% | 1.72% | 32.088 | 13.596 | £33,363 | £40,305 |

| **ISS 9 to 15 = £733, ISS 16+ = £1409.50** | | | | | | | | | |
| --- | --- | --- | --- | --- | --- | --- | --- | --- | --- |
| **Strategy** | **Probability of been sent to the MTC** | **Probability of been sent to the MTC (ISS ≥ 16)** | **Probability of been sent to the MTC (ISS < 16)** | **Proportion of patients who died before discharge** | **Proportion of patients who die between discharge and 1-year post-injury** | **Mean years lived** | **Mean discounted QALYs** | **Mean discounted Costs** | **ICER** |
| **28.4% Sens, 88.6% Spec** | 18.4% | 51.7% | 15.2% | 4.78% | 1.78% | 32.053 | 13.580 | £32,943 |  |
| **57.0% Sens, 80.0% Spec** | 27.7% | 71.0% | 23.4% | 4.72% | 1.76% | 32.067 | 13.586 | £33,066 | £19,730 |
| **64.2% Sens, 76.1% Spec** | 31.5% | 75.9% | 27.2% | 4.70% | 1.75% | 32.070 | 13.588 | £33,098 | £20,816 |
| **69.8% Sens, 70.1% Spec** | 37.1% | 79.6% | 32.9% | 4.69% | 1.75% | 32.073 | 13.589 | £33,127 | ED |
| **74.6% Sens, 65.7% Spec** | 41.2% | 82.9% | 37.1% | 4.68% | 1.74% | 32.075 | 13.590 | £33,150 | ED |
| **87.5% Sens, 62.8% Spec** | 44.5% | 91.6% | 39.9% | 4.65% | 1.73% | 32.082 | 13.593 | £33,205 | £21,012 |
| **90.4% Sens, 58.4% Spec** | 48.5% | 93.5% | 44.1% | 4.65% | 1.73% | 32.083 | 13.594 | £33,221 | £24,780 |
| **94.8% Sens, 18.7% Spec** | 83.4% | 96.5% | 82.1% | 4.64% | 1.72% | 32.085 | 13.594 | £33,276 | ED |
| **99.8% Sens, 2.5% Spec** | 97.8% | 99.9% | 97.6% | 4.62% | 1.72% | 32.088 | 13.596 | £33,311 | £44,694 |

| **ISS 9 to 15 = £366.50, ISS 16+ = £1409.50** | | | | | | | | | |
| --- | --- | --- | --- | --- | --- | --- | --- | --- | --- |
| **Strategy** | **Probability of been sent to the MTC** | **Probability of been sent to the MTC (ISS ≥ 16)** | **Probability of been sent to the MTC (ISS < 16)** | **Proportion of patients who died before discharge** | **Proportion of patients who die between discharge and 1-year post-injury** | **Mean years lived** | **Mean discounted QALYs** | **Mean discounted Costs** | **ICER** |
| **28.4% Sens, 88.6% Spec** | 18.4% | 51.7% | 15.2% | 4.78% | 1.78% | 32.053 | 13.580 | £32,934 | **-** |
| **57.0% Sens, 80.0% Spec** | 27.7% | 71.0% | 23.4% | 4.72% | 1.76% | 32.067 | 13.586 | £33,053 | £19,019 |
| **64.2% Sens, 76.1% Spec** | 31.5% | 75.9% | 27.2% | 4.70% | 1.75% | 32.070 | 13.588 | £33,084 | £19,515 |
| **69.8% Sens, 70.1% Spec** | 37.1% | 79.6% | 32.9% | 4.69% | 1.75% | 32.073 | 13.589 | £33,109 | ED |
| **74.6% Sens, 65.7% Spec** | 41.2% | 82.9% | 37.1% | 4.68% | 1.74% | 32.075 | 13.590 | £33,130 | ED |
| **87.5% Sens, 62.8% Spec** | 44.5% | 91.6% | 39.9% | 4.65% | 1.73% | 32.082 | 13.593 | £33,183 | £19,403 |
| **90.4% Sens, 58.4% Spec** | 48.5% | 93.5% | 44.1% | 4.65% | 1.73% | 32.083 | 13.594 | £33,197 | £21,209 |
| **94.8% Sens, 18.7% Spec** | 83.4% | 96.5% | 82.1% | 4.64% | 1.72% | 32.085 | 13.594 | £33,231 | ED |
| **99.8% Sens, 2.5% Spec** | 97.8% | 99.9% | 97.6% | 4.62% | 1.72% | 32.088 | 13.596 | £33,258 | £30,282 |

| **ISS 9 to 15 = £1541, ISS 16+ = £704.75** | | | | | | | | | |
| --- | --- | --- | --- | --- | --- | --- | --- | --- | --- |
| **Strategy** | **Probability of been sent to the MTC** | **Probability of been sent to the MTC (ISS ≥ 16)** | **Probability of been sent to the MTC (ISS < 16)** | **Proportion of patients who died before discharge** | **Proportion of patients who die between discharge and 1-year post-injury** | **Mean years lived** | **Mean discounted QALYs** | **Mean discounted Costs** | **ICER** |
| **28.4% Sens, 88.6% Spec** | 18.4% | 51.7% | 15.2% | 4.78% | 1.78% | 32.053 | 13.580 | £32,928 |  |
| **57.0% Sens, 80.0% Spec** | 27.7% | 71.0% | 23.4% | 4.72% | 1.76% | 32.067 | 13.586 | £33,049 | £19,359 |
| **64.2% Sens, 76.1% Spec** | 31.5% | 75.9% | 27.2% | 4.70% | 1.75% | 32.070 | 13.588 | £33,083 | £21,714 |
| **69.8% Sens, 70.1% Spec** | 37.1% | 79.6% | 32.9% | 4.69% | 1.75% | 32.073 | 13.589 | £33,116 | ED |
| **74.6% Sens, 65.7% Spec** | 41.2% | 82.9% | 37.1% | 4.68% | 1.74% | 32.075 | 13.590 | £33,142 | ED |
| **87.5% Sens, 62.8% Spec** | 44.5% | 91.6% | 39.9% | 4.65% | 1.73% | 32.082 | 13.593 | £33,195 | £22,061 |
| **90.4% Sens, 58.4% Spec** | 48.5% | 93.5% | 44.1% | 4.65% | 1.73% | 32.083 | 13.594 | £33,215 | £30,748 |
| **94.8% Sens, 18.7% Spec** | 83.4% | 96.5% | 82.1% | 4.64% | 1.72% | 32.085 | 13.594 | £33,313 | ED |
| **99.8% Sens, 2.5% Spec** | 97.8% | 99.9% | 97.6% | 4.62% | 1.72% | 32.088 | 13.596 | £33,364 | £74,478 |

| **ISS 9 to 15 = £1541, ISS 16+ = £704.75** | | | | | | | | | |
| --- | --- | --- | --- | --- | --- | --- | --- | --- | --- |
| **Strategy** | **Probability of been sent to the MTC** | **Probability of been sent to the MTC (ISS ≥ 16)** | **Probability of been sent to the MTC (ISS < 16)** | **Proportion of patients who died before discharge** | **Proportion of patients who die between discharge and 1-year post-injury** | **Mean years lived** | **Mean discounted QALYs** | **Mean discounted Costs** | **ICER** |
| **28.4% Sens, 88.6% Spec** | 18.4% | 51.7% | 15.2% | 4.78% | 1.78% | 32.053 | 13.580 | £32,927 |  |
| **57.0% Sens, 80.0% Spec** | 27.7% | 71.0% | 23.4% | 4.72% | 1.76% | 32.067 | 13.586 | £33,047 | £19,214 |
| **64.2% Sens, 76.1% Spec** | 31.5% | 75.9% | 27.2% | 4.70% | 1.75% | 32.070 | 13.588 | £33,080 | £21,451 |
| **69.8% Sens, 70.1% Spec** | 37.1% | 79.6% | 32.9% | 4.69% | 1.75% | 32.073 | 13.589 | £33,112 | ED |
| **74.6% Sens, 65.7% Spec** | 41.2% | 82.9% | 37.1% | 4.68% | 1.74% | 32.075 | 13.590 | £33,138 | ED |
| **87.5% Sens, 62.8% Spec** | 44.5% | 91.6% | 39.9% | 4.65% | 1.73% | 32.082 | 13.593 | £33,191 | £21,781 |
| **90.4% Sens, 58.4% Spec** | 48.5% | 93.5% | 44.1% | 4.65% | 1.73% | 32.083 | 13.594 | £33,210 | £30,012 |
| **94.8% Sens, 18.7% Spec** | 83.4% | 96.5% | 82.1% | 4.64% | 1.72% | 32.085 | 13.594 | £33,304 | ED |
| **99.8% Sens, 2.5% Spec** | 97.8% | 99.9% | 97.6% | 4.62% | 1.72% | 32.088 | 13.596 | £33,354 | £71,532 |

| **ISS 9 to 15 = £1541, ISS 16+ = £704.75** | | | | | | | | | |
| --- | --- | --- | --- | --- | --- | --- | --- | --- | --- |
| **Strategy** | **Probability of been sent to the MTC** | **Probability of been sent to the MTC (ISS ≥ 16)** | **Probability of been sent to the MTC (ISS < 16)** | **Proportion of patients who died before discharge** | **Proportion of patients who die between discharge and 1-year post-injury** | **Mean years lived** | **Mean discounted QALYs** | **Mean discounted Costs** | **ICER** |
| **28.4% Sens, 88.6% Spec** | 18.4% | 51.7% | 15.2% | 4.78% | 1.78% | 32.053 | 13.580 | £32,918 |  |
| **57.0% Sens, 80.0% Spec** | 27.7% | 71.0% | 23.4% | 4.72% | 1.76% | 32.067 | 13.586 | £33,034 | £18,501 |
| **64.2% Sens, 76.1% Spec** | 31.5% | 75.9% | 27.2% | 4.70% | 1.75% | 32.070 | 13.588 | £33,065 | £20,149 |
| **69.8% Sens, 70.1% Spec** | 37.1% | 79.6% | 32.9% | 4.69% | 1.75% | 32.073 | 13.589 | £33,094 | ED |
| **74.6% Sens, 65.7% Spec** | 41.2% | 82.9% | 37.1% | 4.68% | 1.74% | 32.075 | 13.590 | £33,118 | ED |
| **87.5% Sens, 62.8% Spec** | 44.5% | 91.6% | 39.9% | 4.65% | 1.73% | 32.082 | 13.593 | £33,169 | £20,425 |
| **90.4% Sens, 58.4% Spec** | 48.5% | 93.5% | 44.1% | 4.65% | 1.73% | 32.083 | 13.594 | £33,186 | £26,440 |
| **94.8% Sens, 18.7% Spec** | 83.4% | 96.5% | 82.1% | 4.64% | 1.72% | 32.085 | 13.594 | £33,259 | ED |
| **99.8% Sens, 2.5% Spec** | 97.8% | 99.9% | 97.6% | 4.62% | 1.72% | 32.088 | 13.596 | £33,301 | £57,120 |

| **ISS 9 to 15 = £1541, ISS 16+ = £704.75** | | | | | | | | | |
| --- | --- | --- | --- | --- | --- | --- | --- | --- | --- |
| **Strategy** | **Probability of been sent to the MTC** | **Probability of been sent to the MTC (ISS ≥ 16)** | **Probability of been sent to the MTC (ISS < 16)** | **Proportion of patients who died before discharge** | **Proportion of patients who die between discharge and 1-year post-injury** | **Mean years lived** | **Mean discounted QALYs** | **Mean discounted Costs** | **ICER** |
| **28.4% Sens, 88.6% Spec** | 18.4% | 51.7% | 15.2% | 4.78% | 1.78% | 32.053 | 13.580 | £32,910 |  |
| **57.0% Sens, 80.0% Spec** | 27.7% | 71.0% | 23.4% | 4.72% | 1.76% | 32.067 | 13.586 | £33,021 | £17,790 |
| **64.2% Sens, 76.1% Spec** | 31.5% | 75.9% | 27.2% | 4.70% | 1.75% | 32.070 | 13.588 | £33,051 | £18,854 |
| **69.8% Sens, 70.1% Spec** | 37.1% | 79.6% | 32.9% | 4.69% | 1.75% | 32.073 | 13.589 | £33,077 | ED |
| **74.6% Sens, 65.7% Spec** | 41.2% | 82.9% | 37.1% | 4.68% | 1.74% | 32.075 | 13.590 | £33,098 | ED |
| **87.5% Sens, 62.8% Spec** | 44.5% | 91.6% | 39.9% | 4.65% | 1.73% | 32.082 | 13.593 | £33,147 | £19,066 |
| **90.4% Sens, 58.4% Spec** | 48.5% | 93.5% | 44.1% | 4.65% | 1.73% | 32.083 | 13.594 | £33,162 | £22,853 |
| **94.8% Sens, 18.7% Spec** | 83.4% | 96.5% | 82.1% | 4.64% | 1.72% | 32.085 | 13.594 | £33,215 | ED |
| **99.8% Sens, 2.5% Spec** | 97.8% | 99.9% | 97.6% | 4.62% | 1.72% | 32.088 | 13.596 | £33,248 | £42,713 |

| **ISS 9 to 15 = £1541, ISS 16+ = £704.75** | | | | | | | | | |
| --- | --- | --- | --- | --- | --- | --- | --- | --- | --- |
| **Strategy** | **Probability of been sent to the MTC** | **Probability of been sent to the MTC (ISS ≥ 16)** | **Probability of been sent to the MTC (ISS < 16)** | **Proportion of patients who died before discharge** | **Proportion of patients who die between discharge and 1-year post-injury** | **Mean years lived** | **Mean discounted QALYs** | **Mean discounted Costs** | **ICER** |
| **28.4% Sens, 88.6% Spec** | 18.4% | 51.7% | 15.2% | 4.78% | 1.78% | 32.053 | 13.580 | £32,902 |  |
| **57.0% Sens, 80.0% Spec** | 27.7% | 71.0% | 23.4% | 4.72% | 1.76% | 32.067 | 13.586 | £33,009 | £17,077 |
| **64.2% Sens, 76.1% Spec** | 31.5% | 75.9% | 27.2% | 4.70% | 1.75% | 32.070 | 13.588 | £33,036 | £17,559 |
| **69.8% Sens, 70.1% Spec** | 37.1% | 79.6% | 32.9% | 4.69% | 1.75% | 32.073 | 13.589 | £33,059 | ED |
| **74.6% Sens, 65.7% Spec** | 41.2% | 82.9% | 37.1% | 4.68% | 1.74% | 32.075 | 13.590 | £33,078 | ED |
| **87.5% Sens, 62.8% Spec** | 44.5% | 91.6% | 39.9% | 4.65% | 1.73% | 32.082 | 13.593 | £33,126 | £17,708 |
| **90.4% Sens, 58.4% Spec** | 48.5% | 93.5% | 44.1% | 4.65% | 1.73% | 32.083 | 13.594 | £33,138 | £19,282 |
| **94.8% Sens, 18.7% Spec** | 83.4% | 96.5% | 82.1% | 4.64% | 1.72% | 32.085 | 13.594 | £33,170 | ED |
| **99.8% Sens, 2.5% Spec** | 97.8% | 99.9% | 97.6% | 4.62% | 1.72% | 32.088 | 13.596 | £33,195 | £28,301 |

# References

1. Bouamra O, Wrotchford A, Hollis S, Vail A, Woodford M, Lecky F. Outcome prediction in trauma. Injury. 2006;

2. Bouamra O, Jacques R, Edwards A, Yates DW, Lawrence T, Jenks T, et al. Prediction modelling for trauma using comorbidity and “true” 30-day outcome. Emerg Med J. 2015;

3. Moran CG, Lecky F, Bouamra O, Lawrence T, Edwards A, Woodford M, et al. Changing the System - Major Trauma Patients and Their Outcomes in the NHS (England) 2008–17. EClinicalMedicine [Internet]. 2018 Aug;2–3:13–21. Available from: https://linkinghub.elsevier.com/retrieve/pii/S2589537018300075

4. Newgard CD, Yang Z, Nishijima D, McConnell KJ, Trent SA, Holmes JF, et al. Cost-effectiveness of field trauma triage among injured adults served by emergency medical services. J Am Coll Surg. 2016;

5. MacKenzie EJ, Rivara FP, Jurkovich GJ, Nathens AB, Frey KP, Egleston BL, et al. A National Evaluation of the Effect of Trauma-Center Care on Mortality. N Engl J Med. 2006;354:366–78.

6. Davidson GH, Hamlat CA, Rivara FP, Koepsell TD, Jurkovich GJ, Arbabi S. Long-term survival of adult trauma patients. JAMA - J Am Med Assoc. 2011;

7. Cameron CM, Purdie DM, Kliewer E V., McClure RJ. Ten-year outcomes following traumatic brain injury: A population-based cohort. Brain Inj. 2008;

8. W. A, R. A, Ahmed W, Alwe R, Wade D. One-year functional outcomes following major trauma: experience of a UK level 1 major trauma centre. Clin Rehabil [Internet]. 2017;31(12):1646–52. Available from: http://ovidsp.ovid.com/ovidweb.cgi?T=JS&CSC=Y&NEWS=N&PAGE=fulltext&D=med13&AN=28580790 http://resolver.shef.ac.uk/?http://find.shef.ac.uk/openurl/44SFD/44SFD_services_page?sid=OVID:medline&id=pmid:28580790&id=doi:10.1177%2F0269215517712044&issn=0269-2155&

9. Ara R, Brazier JE. Populating an economic model with health state utility values: Moving toward better practice. Value Heal. 2010;

10. NHS Improvement. 2017/18 and 2018/19 National Tariff Payment System. Online Source Available from https//improvement.nhs.uk/documents/1044/2017-18_and_2018-19_National_Tariff_Payment_System.pdf. 2019;(Last Accessed: 19th July 2019).

11. Christensen MC, Ridley S, Lecky FE, Munro V, Morris S. Outcomes and costs of blunt trauma in England and Wales. Crit Care. 2008;

12. Christensen MC, Nielsen TG, Ridley S, Lecky FE, Morris S. Outcomes and costs of penetrating trauma injury in England and Wales. Injury. 2008;

13. NHS Improvement. 2017/18 reference cost data. Online Source Available from https//improvement.nhs.uk/resources/reference-costs/. 2019;(Last Accessed 19th July 2019).

14. Cameron CM, Purdie DM, Kliewer E V., McClure RJ. Ten-year health service use outcomes in a population-based cohort of 21 000 injured adults: The Manitoba Injury Outcome Study. Bull World Health Organ. 2006;

15. Tappenden P, Chilcott J, Brennan A, Squires H, Stevenson M. Whole disease modeling to inform resource allocation decisions in cancer: A methodological framework. Value Heal. 2012;
